# Green Tea Quality Evaluation Based on Its Catechins and Metals Composition in Combination with Chemometric Analysis

**DOI:** 10.3390/molecules23071689

**Published:** 2018-07-11

**Authors:** Wojciech Koch, Wirginia Kukula-Koch, Łukasz Komsta, Zbigniew Marzec, Wojciech Szwerc, Kazimierz Głowniak

**Affiliations:** 1 Chair and Department of Food and Nutrition, Medical University of Lublin, 4a Chodźki Str., 20-093 Lublin, Poland; zbigniew.marzec@umlub.pl; 2 Chair and Department of Pharmacognosy with Medical Plant Unit, Medical University of Lublin, 1 Chodźki Str., 20-093 Lublin, Poland; virginia.kukula@gmail.com (W.K.-K.); kglowniak@pharmacognosy.org (K.G.); 3 Department of Medicinal Chemistry, Medical University of Lublin, 4 Jaczewskiego str., 20-090 Lublin, Poland; lukasz.komsta@umlub.pl; 4 Department of Analytical Chemistry, Medical University of Lublin, 4a Chodźki Str., 20-093 Lublin, Poland; wojciech.szwerc@umlub.pl; 5 Department of Cosmetology, University of Information Technology and Management in Rzeszów, Kielnarowa 386a, 36-020 Tyczyn, Poland

**Keywords:** green teas, catechins, metals, LC-ESI-Q-TOF-MS, DPPH, ABTS

## Abstract

Green tea infusions are one of the most popular beverages consumed across the world, especially is Asian countries. Green tea quality is primarily based on catechin content, however, the concentration of elements could also significantly influence its biological properties and thus quality and safety. The main purpose of the present study was the evaluation of catechin, antioxidant activity and metal content (K, Na, Ca, Mg, Fe, Mn, Cu, Zn, Cr, Pb, Cd and Ni) in different green tea types cultivated in Japan, Sri Lanka, South Korea, India, China and Japan. The chemical analysis of samples was performed using LC-ESI-Q-TOF-MS for organic constituents and atomic absorption spectrometry (flame and electrothermal) method for inorganic ones. The obtained results were subjected to chemometric elaboration. EGC (213 mg/100 mL of the tea infusion in South Korean Jeoncha) and EGCG (124 mg/100 mL in Japanese Sencha) were the dominant catechins in all green tea samples. Chinese and Indian green tea samples contained the highest concentration of toxic heavy metals, however these values were far below appropriate limitations for green teas. PCA revealed significant similarities between Japanese samples and Korean Jeoncha. In general the latter one was evaluated to have the best quality based on the investigated parameters.

## 1. Introduction

A recently published WHO report revealed that noncommunicable diseases (NCDs), such as cardio- and cerebral diseases, cancers, endocrine/metabolic diseases, including type 2 diabetes and obesity, account for 38 million deaths worldwide. Moreover the WHO predicts an increase in mortality caused by NCDs to 52 million in 2030 [1,2]. Fast progress of that impairments is mostly associated with improper lifestyle, among which negative nutritional changes, which are observed during last 5–6 decades, are considered as the most significant [3,4,5]. As a result of these changes, the oxidative stress, caused by an increased generation of reactive oxygen species (ROS) occurs more and more frequently, and this mechanism is proposed as a key factor in the development of various NCDs [6,7,8]. Although the changes in lifestyle and diet are still considered as the most significant, many data suggest that increased dietary intake of antioxidants is a crucial factor in the prevention of NCDs [9,10,11,12]. Among many secondary metabolites of plant origin, polyphenols are the most abundant and nutritionally significant ones, possessing a wide spectrum of biological activities [13]. Because of the significant concentration of catechins (flavan-3-ols) and high daily intake (estimated at 120 mL/person/day), tea infusions are recently considered as the major sources of these chemicals for a general population [14,15,16]. Although black tea is the major worldwide type of tea, the green teas are considered as the most active and rich in polyphenols [15,17,18]. Green tea, which accounts for 20% of the global tea production, is a non-fermented product, obtained by harvesting fresh leaves, which are immediately steamed to prevent enzymatic degradation of its active compounds. This tea type is the most common in the Far East, where its consumption has very strong traditional and historical roots [14,19,20]. However, because of its wide spectrum of biological activities, its popularity is still increasing in other parts of the world, including Europe and the USA.

Polyphenolic compounds are the major type of secondary metabolites present in tea. The major fraction of polyphenols in green tea, a main reason of its biological properties, are catechins with simple structures, among which the most important are (−)-epicatechin (EC), (−)-epicatechin gallate (ECG), (−)-epigallocatechin (EGC) and (−)-epigallocatechin-3-gallate (EGCG). Due to the fact that many catechins are esterified with gallic acid (GA), it is a major phenolic acid present in the extracts [21]. Large number of biological properties of *Camellia sinensis* were described in numerous scientific papers, including its antibacterial, antiviral, antioxidant, antiallergenic and cardiovascular or cancer prevention activities [17,22,23,24,25].

Tea contains also complexed mineral composition, including both bio- and toxic elements, such as cadmium, lead and nickel. Because of a high daily intake of tea infusions it can be considered as a significant product in total dietary intake of essential minerals, but also as a potential source of detrimental metal ions for humans [26]. Mineral composition is also a crucial factor used in tea discrimination, regarding its quality, origin, type or technological processing, using different chemometric tools, e.g., principal component analysis (PCA), cluster analysis or linear discriminant analysis (LDA) [27,28]. Moreover, numerous studies have been published dealing with the quality assessment of green teas based on its organic composition, where thorough statistical analysis was used to elucidate obtained results [18,29]. However, no studies on green tea can be found, which uses this wide spectrum of parameters, including catechin composition, metal concentration and antioxidant activity, supported by chemometric elaboration of obtained data in a single investigation, allowing to look into the dataset in multivariate manner and interpret complex trends inside the results.

Therefore the main aim of the study was to evaluate the quality of green teas from different geographical regions, based on their catechin composition, metal concentration, antioxidant properties, expressed as total phenolic content (TPC) and antiradical activity (DPPH and ABTS tests), in combination with chemometric elaboration by PCA. The secondary purpose of this research was to find significant correlations between particular green tea constituents and also discriminate their origin based on their secondary metabolites content, metal concentration, antioxidant properties and combination of all those parameters. Additional aim of this work was to evaluate the significance of green tea infusions as a potential source of bioelements for humans, and also compare heavy metals concentration with appropriate safe level limits. Characterization of the plant material used in the study is presented in Table 1, including special voucher specimen numbers, which were used throughout the whole manuscript in all tables and figures.

## 2. Results and Discussion

### 2.1. Macro- and Trace Elements Content in the Green Tea Samples

The content of the 4 macro- (Na, K, Mg and Ca), 5 trace elements (Mn, Zn, Cu, Fe and Cr) and toxic heavy metals (Cd, Pb and Ni) were presented in Table 2 and Table 3. The study revealed that the highest content was determined for potassium, magnesium, calcium, and manganese. Iron was also a significant element, however, its quantities were much lower compared to these mentioned above. Investigated samples were also characterized by low contents of sodium, zinc and copper. Regarding all studied green teas, the metal content can be ranked in the following order: K>Mg>Ca>Mn>Fe>Na>Zn>Cu>Ni>Cr>Pb>Cd. Potassium was the main element determined in all teas. The highest, as well as the lowest amounts, were found in Japanese green teas, Matcha and Sencha, respectively.

Based on the obtained results, the average ratio of potassium to sodium was calculated as 207:1, which means that these products could constitute an important part of the human diet, especially for the part of the population with cardiovascular problems. Surprisingly, these data are in a good agreement with our previous findings suggesting a very high ratio of potassium to sodium in the case of ginger from ecological plantations [30]. Regarding green teas, this ratio is even six times higher. Taking into account high magnesium content, green tea can be considered as especially healthy product in the prevention of cardiovascular disorders, based only on its mineral composition, even without considering other biological activities. Also, our findings resemble some previous reports, which indicated potassium as the main metal in green teas (average concentration 16,000–25,000 mg/kg) [31,32]. Chu and co-workers [31] reported that the content of magnesium in green teas ranged widely from 1200 to 3000 mg/kg. Similar results were obtained by Konieczyński and co-investigators [33], who revealed magnesium contents in the range of 1060–1610 mg/kg (d.w.) in different green tea samples. Marked differences between the samples were observed in magnesium and calcium concentration, depending on the origin and green tea type used in our study. Chinese samples were characterized by the highest concentrations of these two elements (CH and CH1 were very high in magnesium and calcium, respectively), whereas Japanese green teas contained the lowest amount of Ca (JS) and Mg (JA). Sodium was the macroelement present in the lowest quantity, however, its concentration was still high compared to other data [31]. It noteworthy that Na concentration in green tea is much lower compared to other tea types (black or Pu-erh), which also may play a crucial role, regarding the global sodium intake restrictions [26]. Results of the present study were in a very good agreement with previous findings on sodium content in green tea samples from China and Japan, however, in comparison with the Indian samples our data were significantly higher [27].

Regarding trace elements content and in the light of previous findings, it was expected that the quantity of manganese would belong to the highest and will be followed by iron [27,32,33,34]. However, the concentrations of these elements varied depending on the origin of tea. Chinese and Indian samples contained much higher amounts of Mn and Fe comparing to the teas cultivated in Nepal or South Korea. Brzezicha-Cirocka and co-investigators [27] reported similar values of Mn in Chinese teas and Fe in Indian samples, however, they found higher amounts of Fe in green teas from China, compared to our results (336 mg/100 g). Sencha samples contained the highest quantity of zinc, whereas Matcha was characterized by the lowest concentration of this element. Generally the content of this mineral in teas from different countries was on a similar level, which was close to the previous studies finding zinc concentrations in different types of teas in the narrow range 20–40 mg/kg [27,32,33]. In most countries the level of zinc in tea is not limited by local laws, except Australia where the permissible concentration was set to 50 mg/kg [34]. No sample in the present study exceeded this value. The highest amounts of Cu were found in the Chinese samples (both original and blended ones), which resembled the report of Brzezicha-Cirocka and co-investigators (19.7 mg/kg in green teas from China) [27]. The lowest concentrations of this element were determined in green teas from South Korea and Japan. These results were significantly lower compared to previous reports suggesting that the content of this trace element in green tea can reach even 40 mg/kg [32,35]. Copper is a trace element which content in tea is limited. However permissible limits differ significantly according to countries and organizations and cannot exceed 40 (Germany) [34], 60 (China) [36], 100 (Japan) [37] or 150 mg/kg (Australia, India, Iran, Singapore, United Kingdom or United States) [37,38,39,40]. In the present study the content of Cu in all investigated samples did not exceed 17.9 mg/kg and was far below admissible limits presented above. Chromium is a trace elements, which concentrations in green teas are much lower compared to black teas, mainly because of the usage of the crush-tear-curl rollers during the production process of the latter one. Moreover green tea is produced using young leaves in which the concentration of Cr is lower, compared to black teas, where mostly older plant material is used [40]. In the present study the determined levels of Cr were low and significantly the highest values were found in Japanese Matcha. There are studies suggesting high content of chromium in green tea of Japanese origin (1.6 mg/kg), but majority of similar studies reports Cr content in green tea below 1 mg/kg [27,40,41].

### 2.2. Toxic Heavy Metals Content in Green Tea Samples

Table 4 presents average content of heavy metals in the investigated samples. The content of lead in the investigated samples was very differentiated. In Japanese Sencha the levels were below LOD. The lowest amounts were determined in the teas cultivated in Nepal and Sri Lanca and much higher from South Korea and Japan (Matcha and Agari types). The highest content was measured in products from China (blended samples) and India. Other findings also report lead content in green teas in a very broad spectrum ranging from 90 to even 8300 µg/kg [27,32,35]. Peng and co-investigators [35], based on the results obtained for green teas from 26 plantations, established the average level of Pb of 1.07 mg/kg, but with SD value of 0.54 mg/kg, which shows how differential these values are. Although there are significant discrepancies between permissible limits of Pb in tea set by different countries, data obtained in the present study are far below these limitations (2 mg/kg —Vietnam; 5 mg/kg—Germany; 10 mg/kg—Canada, India, Thailand and WHO recommendations) [37,40,42,43,44].

The mean concentration of cadmium was above 10 µg/kg only for teas from China (blended products) and India. For the rest of the samples, it was below 5 µg/kg. The results were in a good agreement with other data, which established Cd content in green tea in a very wide range 10–100 µg/kg [27,35,40]. The findings of the present study, as well as other authors, confirmed that there is no potential risk regarding Cd content in the green tea, as obtained results are far below limitations established by Germany (0.2 mg/kg), Canada and WHO (0.3 mg/kg) or Vietnam (1 mg/kg) [40,42,43,44].

Regarding nickel concentration in the investigated green teas, the highest values were determined in samples from South Korea, followed by Chinese tea (blended). The content of Ni in the rest of the samples was ≤5 mg/kg. Obtained results are in full agreement with other data reporting Ni concentration in green tea in the very wide range 0.2–22.9 mg/kg, depending on type, origin or even different provinces of the same country [27,32,40]. According to the regulations, the levels of Ni in tea are not limited in majority of countries. To the best of our knowledge the only limitation could by found in India, where the acceptable level of this metal cannot exceed 5 mg/kg [38]. Taking into account this value, the majority of samples in the present study contained nickel within this limitation, except few samples from South Korea, China and Japan (Sencha and Agari). Desideri and co-workers [45] based on the results obtained for teas from different countries established the mean level of Ni at 5.6 mg/kg with the range 3.2–7.1 mg/kg (for green teas). They also concluded very close level of nickel in both black and green tea samples. Similar results were obtained in other study, where determined Ni levels were 4.50–5.21 and 4.13–7.28 mg/kg, in Indian and Ceylon black tea samples respectively [46]. This shows big diversity in the content of Ni in tea leaves, which was proved in the present study.

### 2.3. Estimation of Daily Dietary Intake of Metals with Green Tea

Based on the literature data on green tea consumption and leaching factors the daily intake of metals was established, regarding RDA values for each element [27,32,47]. According to Cao and co-workers [48] the average consumption of non-fermented green tea leaves was established as 3.92 g/day. Regarding the much lower popularity of green tea in Europe, a mean intake of 2 g was proposed, which was in agreement with consumption of 200 mL infusion per day [28]. For toxic heavy metals estimation of dietary risk associated with green tea consumption was established, regarding mean body weight of 60 kg and appropriate tolerable daily intake (TDI) values [27,47]. Calculations were presented in Table 5. Regarding macroelement content, green tea infusinons are not a good source of these metals for general population as the mean contribution to RDA or AI values is far below 1% for Na, K, Mg and Ca. Even in countries were green tea consumption is much higher (according to Peng and co-workers the average green tea consumption in China is 11.4 g/day [35]) it cannot be considered as a significant source of these elements for humans.

In the case of trace elements, green tea infusions are a significant and very important source of Mn, as the consumption of one cup can contribute as much as 1/3 of the daily need for this metal (CH1). This is with agreement with other data suggesting tea as a very important source of Mn for humans [27,32,35,49]. Recent findings of Brzezicha-Cirocka and co-investigators [27] indicated green tea as important source of dietary chromium. Our results confirmed these data as the mean contribution was found to be almost 2% and for some samples even >5% of the daily recommendation for this metal. In the case of other elements green tea should not be considered as significant dietary source of these. Interestingly this product may be a good source of iron, but because of a low leaching factors (mostly insoluble iron salts), its concentration in infusion is scarce.

Dietary exposure to toxic heavy metals is insignificant as the mean exposure to Pb and Cd is far below 1% of TDI. Even for Chinese samples, which contained the highest amounts of these elements the exposure was <0.5% and <0.1% of appropriate norm, for lead and cadmium, respectively. These findings are similar to previous reports, suggesting that there is no risk of high exposure to Pb and Cd via green tea drinking, even in countries where this product is very popular and thus consumption is high there [27,32,35,40]. Regarding Ni, green tea may be significant route of exposure to this heavy metal as for some samples (green tea from South Korea) dietary intake contributed to 6% of TDI value, which is high taking into account the consumption of only one cup per day (200 mL). The mean exposure was found only 2% of TDI and thus the health risk to this metal is still low. However, if the intake of green tea is higher, the exposure increases significantly. Li and co-workers [50] estimated that Ni intake via the consumption of green tea was 0.369 mg/kg b.w./day with first infusion, which accounts to over 7% of the TDI value. However the daily consumption of green tea infusion used in this study was 1250 mL/day, which corresponded to the daily dry tea leaves ingestion of 8 g/person/day, which is very high. Other reports did not reveal any health risk associated with green tea consumption and Ni intake [27,35]. Thus our study is the first indicating a potential dietary exposure to this element, even for populations consuming much lower amounts of green tea, compared to Asian countries.

### 2.4. LC-MS Determination of Catechins and Gallic Acid in Green Tea Samples

The elaborated chromatographic method enabled separation, qualitative and quantitative determination of five catechins (C, EC, EGC, EGCG and ECG) and gallic acid, the main phenolic acid, which esterifies polyphenols in tea. Table 6 presents the composition of green teas investigated in the present study. Based on the obtained results the concentration of catechins and gallic acid can be placed in the following order: EGC>EGCG>ECG>EC>C>GA. EGC and EGCG were the most dominant polyphenols in all investigated green tea samples. These two are undoubtedly the major catechins present in green tea infusions. However, according to different sources, the most abundant one is EGCG [21,51] or EGC [52].

Our results are more consistent with data presented by Khokhar and co-workers [53], which suggest that it is hard to clearly point the major catechin as, both EGC or EGCG are dominant, depending on the green tea product. South Korean Jeoncha was the tea most rich in simple catechins (441 mg/100 mL), it contained the highest amounts of EGC (213 mg/L) from all samples studied within this experiment. This Korean green tea is a type of Sencha, regarding the cultivation and production parameters, thus the composition of these two types of teas is very similar, which was proved in this study. In general Korean Jeoncha and all types of Japanese teas were characterized by the highest concentration of catechins. On the other hand samples cultivated in Nepal, India and China contained similar amounts of these simple phenolics (ca. 350 mg/100 mL), which was much lower compared to the previously described. Although green teas from Sri Lanka were of moderate composition regarding this parameter, surprisingly some samples from this region contained very high concentration of EGCG (139 mg/100 mL), which was the highest value measured in this study for this compound. The mean concentration of EC was similar in all tea extracts and was close to the average value of 38.8 mg/100 mL obtained for all infusions. The amounts of GA were far much lower compared to any catechin measured in this study in all teas. An interesting trend was observed regarding GA concentration—in teas the most rich in catechins (Korean and Japanese samples) the content of gallic acid was much lower compared to other samples (e.g., Chinese, Indian or products from Sri Lanka).

There are not much data on catechin concentration in green tea infusions, as the majority of published research describes the content in dry leaves. Because tea is consumed as the infusion and not a dry product we compared different green teas, regarding the composition of their infusions. In our previous study [15] we have investigated black tea infusions from different geographical origins based on the simple catechins composition. Because catechins are mostly degraded during fermentation process of black tea, which is already well known and was described [15,54,55], their concentration in green tea is much higher. It was confirmed in the present study, as the content of EGCG, ECG and EC was on average 100-times higher. Record and Lane [55] investigated the composition of green and black tea infusions and revealed the first to contain much higher concentration of simple catechins. However, in their study the difference was much lower as compared to the results of the present investigation. They also found EGCG and EGC to be dominant phenolics in both kinds of teas. The concentration of (+)-catechin (C) in black tea is mostly below the detection level, as this compound is decomposed during fermentation process [15,54]. In this study we proved that green tea, in contrast to black tea, contains significant amount of this catechin. Although the sum of all simple catechins was found to be much higher in green tea, the concentration of EGC in some black teas was on the similar level, which suggest that not all catechins are decomposed during production process of this kind of tea and thus it may be considered as a significant source of these non-nutrients for the population. This is with agreement with the previous findings [15,55]. Based on the literature data, the concentration of GA in green teas was found to be similar compared to black teas [15,55].

### 2.5. Antioxidant Activity of the Investigated Green Tea Samples

Table 7 presents the results of antioxidant activity (using DPPH and ABTS radicals) and TPC content of the investigated material.

The results of the antiradical scavenging activity using DPPH and ABTS radicals were in a good agreement and were well correlated with TPC parameter, which was expected taking into consideration the mechanism of these in vitro investigations [10,15]. The samples from Sri Lanka, Nepal and China (original ones) were characterized by the highest antioxidant potential. Obtained data were not correlated with the concentration of EGCG or any other catechin. This fact may bring to a conclusion that not only catechins, but also other flavonoids are responsible for the antioxidant activity of green tea. Antioxidant activity of green tea infusions was much higher compared to black teas, when measured using the same protocol and taking into account the dilution factors [15]. However, the differences were not as high as a simple comparison of catechins’ concentration in these two kinds of products might suggest. Similar conclusions were made previously, pointing both, green and black teas, as significant dietary antioxidant agents [55,56].

The results obtained by the LC-MS method were not well correlated with the results obtained using Folin-Ciocalteau method. This technique confirmed high content of polyphenolic compounds only in the case of samples from Sri Lanka, for which the highest concentration of polyphenols was determined (97.3 mg/100 mL). For other teas, containing high concentration of catechins (e.g., Japanese samples), the results of F-C method were rather low. The reason for such a large discrepancy in the obtained results is the fact that this is rather a technique which measures a total reduction power of the sample, and which can be used rather as the method for antioxidant determinations, than phenolic content [57]. Andjelkovic and co-investigators [58] confirmed weak correlation between chromatographic determinations of phenolics with Folin-Ciocalteu results.

### 2.6. Chemometric Elaboration of the Obtained Data

**Figure 1:**
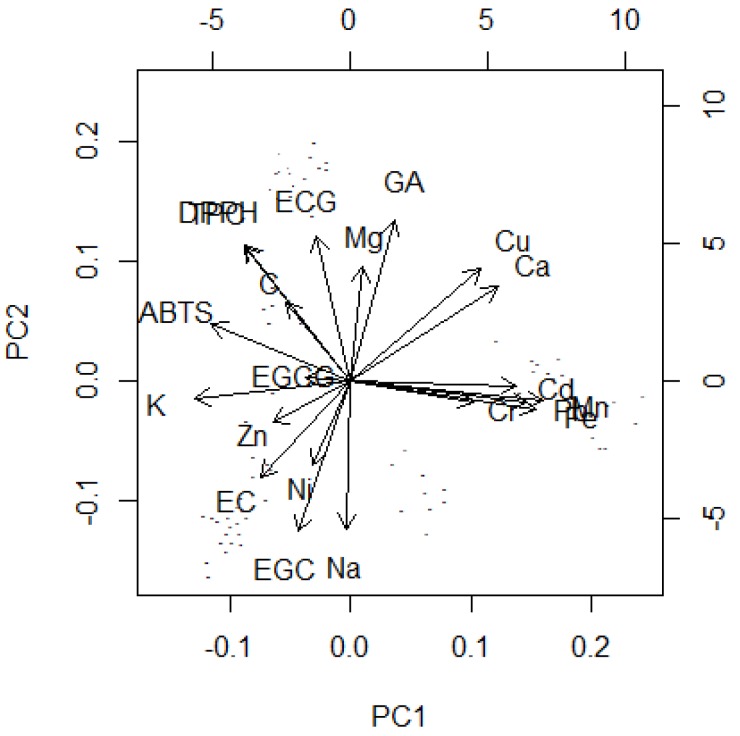

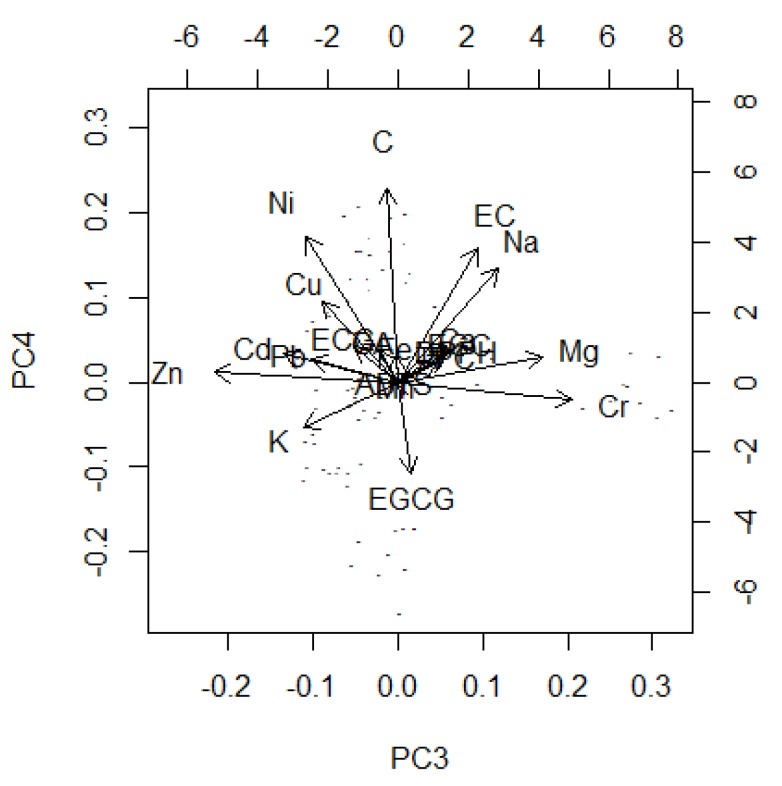
Biplots of Principal Component Analysis for tea dataset. This plot is discussed as loading values plot, scores are represented only as small dots to visualize shape of the dataset. The score plot without loadings, but with annotated teas is presented as Figure 2. GA—gallic acid; C—catechin; EGC—epigallocatechin; EGCG—epigallocatechin-3-gallate; ECG—epicatechin gallate; EC—epicatechin.

**Figure 2:**
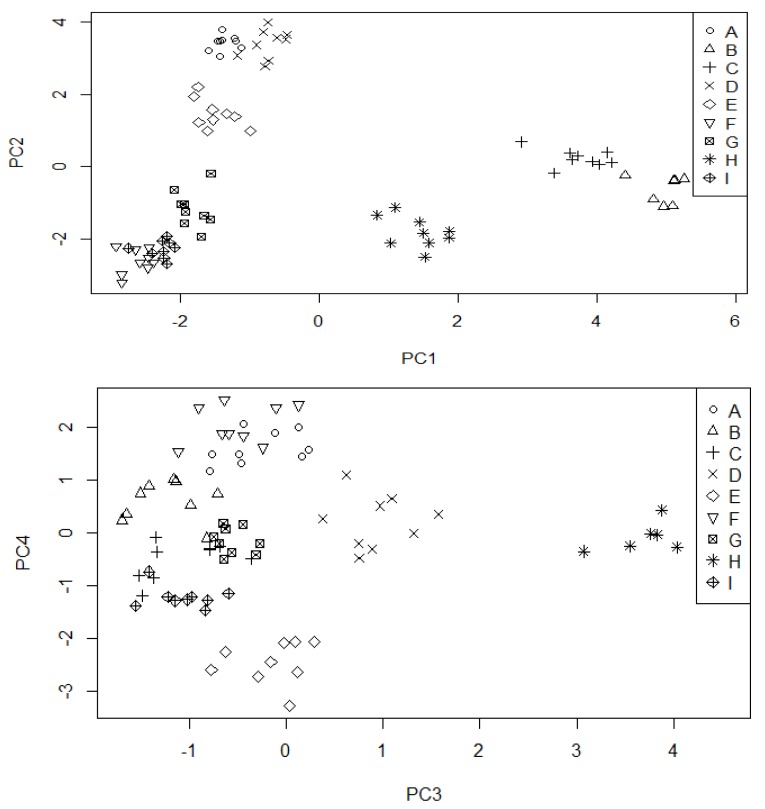
Principal Component Analysis Scores for tea dataset. A—CH (China, original); B—CH1 (China, blended); C—I (India); D—SL (Sri Lanka); E—N (Nepal); F—SKJ (Jeoncha, South Korea); G—JA (Agari, Japan); H—JM (Matcha, Japan); I—JS (Sencha, Japan).

The results were arranged as a matrix with rows (tea samples) and columns (expressing 21 investigated parameters) and subjected to scaled Principal Component Analysis. Obtained results are illustrated in Figure 1 and Figure 2. 80% of total variance was explained by the four first principal components (33.7, 23.5, 11.7 and 8.6%, respectively). This dataset is quite hard to compress and the analysis shows that these parameters are not strictly intercorrelated, so the information is hidden in at least four independent trends. The raw data obtained during PCA analysis were presented in the Dataset S1 (scores and loadings) and Figure S2 (statistical significance of each parameter) in the Supplementary Material.

The first trend (PC1) is mainly connected with differences between Cr, Cd, Mn, Pb and Fe (this group of metals is strictly intercorrelated) and ABTS and K, which are less intercorrelated, but representing the opposite behaviour. CH1 and I tea samples have the largest content of metals and the smallest content of K and ABTS. Matcha is a tea kind which lies in the middle of this trend, whereas the other teas exhibit opposite contents.

The second trend models differences between two intercorrelated (but inversely correlated between themselves) groups: EC, EGC, Na, Ni and Mg, GA, DPPH, TPC. Very high correlation between Cu and Ca should be also noted. Surprisingly, EGCG is not correlated with the first two trends and it is present only in the third and the fourth principal component. Chinese (both original and blended samples) and Nepal are teas with higher values of the first group and lower values of the second. The opposite results can be noted for Japanese and Korean teas: Matcha, Jeoncha and Agari. CH2 tea is located in the middle of PC2 values, however this value is still higher than Japanese and Korean ones.

The two next principal components model relate two smaller trends in the dataset. The third one represents differences of Mg and Cr versus Zn, K and partially Cd and Pb (also intercorrelated). Matcha is quite an outlying tea for large value of this PC (high content of Mg, Cr, low content of Zn, Cd). EGCG is located in the fourth component only, representing almost no positive correlations with any variable, strong negative correlation with C and weaker (but visible) correlation with Ni, Cu, EC and Na. Large values of this parameter can be observed for Nepalese, whereas low values are seen for Jeoncha and Chinese original green tea samples.

## 3. Materials and Methods

### 3.1. Plant Material

Green teas used in the present study were purchased in 2016 from professional tea shops in Poland. Twenty seven different green teas, cultivated in six countries were evaluated. A precise description of the studied material was presented in Table 1, including a voucher specimen number given to each tea. Twenty four products were original, and their origin was guaranteed by the seller. On purpose three types of blended green teas were used, which were commercial products, for which China was indicated as a place of origin (this is marked in the Table 1). The aim of the study was not a comparison between original and blended teas. However the latter were used to check whether their composition markedly differs from original products. Except for these, no blended samples or from an unknown cultivation area were used in the experiment. For each product three different batches were used, and from each batch three different samples were taken. All studies were made in triplicate. In total 243 green tea samples were investigated (9 types × 3 representatives × 3 batch number purchased × 3 samples taken).

### 3.2. Chemicals

All reagents used for metal analysis—nitric (V) acid (65%) and hydrochloric acid (36%)—were of Suprapur Grade and purchased from Merck (Darmstadt, Germany). Certified stock solutions of the investigated elements (1000 mg/L, Merck) were used to plot calibration curves for each metal. An Ultrapure Millipore Direct-Q-R 3UV system (Merck) allowed us to obtain high purity deionized water (resistivity 18.2 MWcm), which was used during all spectroscopic analysis (including the digestion process). Polypropylene tubes were used to store the digests obtained during metal analysis.

The solvents used during LC-MS determinations (acetonitrile, formic acid and water) were of spectroscopic grade and were purchased from J. T. Baker (Center Valley, PA, USA). Standards of catechins (C, EGC, EGCG, ECG, and EC), gallic acid, DPPH (2,2-diphenyl-1-picrylhydrazyl), ABTS [2,2′-Azino-bis(3-ethylbenzothiazoline-6-sulfonic acid)] diammonium salt) and trolox were bought from Sigma-Aldrich (St. Louis, MO, USA). Folin-Ciocalteu reagent, sodium carbonate and citric acid were bought from Stanlab (Lublin, Poland), whereas potassium persulfate (analytical grade) from POCH Polish Chemicals Reagents (Gliwice, Poland).

### 3.3. Metal Analysis of the Samples

#### 3.3.1. Sample Preparation

Each batch of tea was first ground in a ceramic mortar and then three 10 g samples were weighted into quartz crucibles. Although investigated material was dry, the crucibles were placed in an electrical dryer and dried in 105 °C until constant weight was obtained. This step was performed to be sure that the teas were totally dry (determined moisture was below 7% for all samples). Simultaneously six samples of the reference material (25 g each) were weighted into crucibles and subjected to the same protocol as the investigated samples.

#### 3.3.2. Digestion Process

Digestion and metal determinations were performed according to the previously described protocol [30]. Although the method was already validated, during the present study it was once again checked for its accuracy in metal analysis of green tea samples. A mixture of flour and milk powder (70:30), enriched with known amounts of investigated elements was used as a reference material. The results were presented in Table 8. Briefly the samples were ashed in the muffle furnace in 450 °C. The remains of the organic matrix were later oxidized using 30% solution of nitric (V) acid, which was then evaporated and the samples were re-heated in 250 °C during 2 h. After all samples were totally digested the ash was dissolved in 10% hydrochloric acid, filtered and diluted with deionized water to a final volume of 25 mL.

#### 3.3.3. Analytical Determinations Using Flame—(FAAS) and Electrothermal Atomic Absorption Spectrometry (ETAAS) Methods

The concentration of Na, K, Ca, Mg, Mn, Zn, Cu and Fe was measured using FAAS method directly from the digests using SOLAAR M5 apparatus (Thermo Scientific, Waltham, MA, USA) and appropriate dilutions if needed. During calcium determinations, spectral buffer was used (Lanthanum trichloride).

The content of Cr, Ni, Cd and Pb was determined using ETAAS method in a L’vov platform graphite cuvette using a High-Resolution Continuum Source Atomic Absorption Spectrometer ContrAA 700 (Analytik, Jena, Germany). For cadmium, lead, nickel and chromium stock solutions of 5; 60; 60 and 50 ppb were used, respectively. Twenty-five microliters of each digest was mixed with five microliters of matrix modifier [(Pd(NO_3_)_2_/Mg(NO_3_)_2_—if necessary] and injected to the furnace. Calibration curves were plotted based on the decomposition of residue methods and dilutions were performed automatically by the autosampler.

### 3.4. Preparation of Green Tea Infusions

An aqueous infusion was prepared according to previously described methods from each grinded portion of tea, which indicated that when using hot (temperature 80–90 °C), distilled water, the extraction efficiency of catechins is significantly higher compared boiling or cold tap water [52,59]. According to Lin and co-workers [52] the higher the ratio of water to tea leaves, the higher the extraction efficiency. Taking these data into consideration, 1 g of green tea leaves was brewed for 5 min in closed glass vessels using 100 mL of hot distilled water. Subsequently the pH of the infusion was decreased to 3.2, using citric acid (only for LC-MS determinations), as described previously [15], and the brews were made up to a final volume of 100 mL with distilled water. Before the chromatographic analysis the samples were filtered using 0.45 µm filter (Cronus, Gloucester, UK) and diluted 5-times with distilled water.

### 3.5. LC-ESI-Q-TOF-MS Analysis of Green Tea Infusions

Chromatographic analysis was performed using ESI-Q-TOF-MS 6500 Series mass spectrometer (Agilent Technologies, Santa Clara, CA, USA) in combination with an LC system (1200 Series, Agilent Technologies). The set was equipped with a degasser (G1322A), a binary pump (G1312C), a PDA detector (G1315D) and an autosampler (G1329B). The separation of catechins was made using Zorbax RP 18 column (150 mm × 2.1 mm, dp = 3.5 µm) also purchased from Agilent Technologies. The chromatographic method was previously optimized and validated for the determination of catechins in the black tea infusions and described elsewhere [15]. In general, the mobile phase was composed of 0.1% of formic acid in water (solvent A) and 0.1% of formic acid in acetonitrile (solvent B). The introduced gradient of solvents was as follows: 0 min—90% A; 10 min—60% A; 17 min—5% A; 20 min—90% A. The analysis time was set at 30 min and the flow rate at 0.2 mL/min throughout the run. The mass spectra were recorded according to the previously described protocol [15] using a capillary voltage of 4.0 kV, gas and sheath gas temperatures of 350 and 400 °C, the fragmentation voltage of 130 V and the CID values of 10 and 20 V. The collection of spectra was performed in a data dependent method, so as two most intensive signals at a given scan were fragmented in two collision energies (CID values of 10 and 20 V); the above fragmented signals were later excluded for the following 0.5 min to let provide the fragmentation of other less intensive signals.

The identification of all compounds was made based on their retention times and mass spectra (in comparison with the standards, literature data and on-line mass libraries). The quantitative analysis was performed using calibration curves plotted for each investigated compound, which were composed of 7 different concentrations of a standard. The chromatograms obtained during the study were shown in Figure S1 and the MS/MS data in Table S1 (Supplementary Materials). Selected validation parameters for (+)-catechin (not described by the authors in the previous studies) were as follows: LOD: 0.42 ng/mL, LOQ: 1.26 ng/mL, linearity range: 0.010–45 µg/mL.

### 3.6. Evaluation of Antioxidant Activity of Green Tea Infusions

#### 3.6.1. DPPH Test

Antiradical scavenging activity using DPPH radical was performed according to a modified method described elsewhere [15,60]. Green tea infusions were diluted with distilled water (1 + 1 *v*/*v*) and 0.1 mL of each was mixed with 3.9 mL of methanolic solution of DPPH (6 × 10^−5^ M). After the reaction reached the *plateau* value (AC_t_) (max. after 30 min) the absorbance was read at a wavelength of 515 nm in 1-cm cuvettes using UV-Vis spectrophotometer (Thermo Fisher Scientific Evolution, Waltham, MA, USA). Blank samples were prepared by replacing the extracts with methanol. Antioxidant activity of infusions was calculated as a percent of radicals scavenged by the samples, using the following equation: I (inhibition) [%] = [(AC_0_ − AC_t_)/AC_0_] * 100, where AC_0_ represents initial absorbance of DPPH.

#### 3.6.2. ABTS Test

The study was performed according to a protocol described previously by Re and co-workers [61]. Initially 0.36 g of ABTS was dissolved in a small amount of distilled water. In the next step 0.0662 g of potassium persulfate (K_2_S_2_O_8_) was added and after it was totally dissolved the mixture was filled up with water to a final volume of 100 mL. The mixture was left in the dark at room temperature for 16 h to produce ABTS radical cation (ABTS^+^). Then, the solution was diluted 100 times with ethanol. Green tea infusions were diluted with water (1 + 3 *v*/*v*). Next, 10 µL of the samples were mixed with 1 mL of ABTS^+^, the mixture was incubated in the dark at room temperature for 6 min and the absorbance was measure at a wavelength of 736 nm. The antioxidant potential was expressed using Trolox equivalents. For this purpose several concentrations of Trolox in water were prepared (0–15 mM/L) and were used according to the same protocol as investigated samples.

#### 3.6.3. TPC

The modified protocol with Folin-Ciocalteu (F-C) reagent was used according to the method described previously [15]. Each infusion (0.5 mL) was mixed with distilled water and F-C reagent. The pH of a solution was alkalized using 20% Na_2_CO_3_ and after incubation (2 h) the absorbance was read at 760 nm. Simultaneously the calibration curve was performed using gallic acid (concentration range 50–500 mg/L). TPC was calculated as GAE per 100 mL of tea infusion (gallic acid equivalents).

#### 3.6.4. Statistical Analysis

Principal Component Analysis of the obtained results were done in GNU R computational environment, version 3.4.0 running on a Windows 64-bit platform. Built-in function “prcomp” was used without any additional packages.

## 4. Conclusions

A large number of samples tested with complex matrix of parameters in combination with PCA analysis enabled to draw interesting conclusions on the investigated material. Korean and Japanese green tea samples contained the highest amounts of catechins, whereas green teas from Nepal, India and China were characterized by the lowest concentration of these secondary metabolites. Only in a tea sample originating from China (original) and Nepal, EGCG was the main catechin. The highest concentration of EGCG was determined in the infusions form Japanese Sencha and green teas from Sri Lanka. The latter ones were characterized by the highest antioxidant potential. In the rest of the samples the dominant catechin was EGC. In general these were the principal catechins in all tested products. PCA revealed strong negative correlation between C and EGCG content. Moreover visible, significant positive correlation between Cu and Ca concentration in the investigated samples was also proved.

Korean Jeoncha tea contained the highest quantity of polyphenols and clearly the highest content of nickel, whereas Japanese Matcha was characterized by the highest concentration of chromium (far above the rest of the investigated green teas). The latter one may be considered as significant dietary source of chromium for the general population. Chinese teas (both original and blended) contained the highest amount of copper. In general Chinese and Indian green tea samples contained the highest concentration of toxic heavy metals. However these values were far below appropriate norms for green teas. Because of a high content of nickel in some of the investigated samples (Jeoncha and some of the Chinese samples), green tea may be important source of this element, even for population where the consumption of this product is low.

Chemometric elaboration of data did not reveal significant geographical diversity of teas. However, the performed PCA analysis showed that, based on the investigated parameters, two similar groups of teas can be distinguished—Chinese and Nepal teas (first group) and South Korean Jeoncha and Japanese Matcha, Agari and Sencha (second group). All blended tea samples were characterized by a similar composition, regarding their phenolic profile and metals’ content, to original products investigated in the present study.

Taking into account all obtained data, Jeoncha may be considered to have the best quality from all investigated green teas within the present study, followed by Japanese green tea samples.

## Figures and Tables

**Table 1:** Characteristic of the investigated green teas.

Voucher Specimen Number	Sampling Region/Type	Origin	Form	Number (Representatives)
CH	China	Original	loose	3
I	India	Original	loose	3
SL	Sri Lanka	Original	loose	3
N	Nepal	Original	loose	3
SKJ	South Korea/Jeoncha	Original	loose	3
JA	Japan/Agari	Original	loose	3
JM	Japan/Matcha	Original	loose	3
JS	Japan/Sencha	Original	loose	3
CH1	China	Blended	bags	3

**Table 2:** The average content of macroelements in green tea samples according to their origin (including tea type or form).

Parameter	Macroelements (mg/kg)			
	Na	K	Mg	Ca
CH (China) *				
Concentration	49.5 ± 4.34	15,483 ± 877	2115 ± 121	1636 ± 90.3
Range	45.1–57.9	13,783–16,335	1983–2309	1524–1794
CH1 (China) *				
Concentration	66.4 ± 7.68	11,504 ± 1370	1733 ± 96.0	2439 ± 81.3
Range	51.2–74.9	9486–13,737	1521–1801	2314–2542
I (India) *				
Concentration	55.6 ± 4.92	11,050 ± 719	1536 ± 75.9	2440 ± 143
Range	49.1–62.5	9658–11,753	1467–1703	2274–2744
SL (Sri Lanka) *				
Concentration	41.7 ± 1.75	12,975 ± 1849	1999 ± 103	2639 ± 112
Range	38.6–43.7	9290–15,408	1860–2106	2474–2839
N (Nepal) *				
Concentration	18.3 ± 3.08	16,922 ± 994	1981 ± 153	1327 ± 65.8
Range	14.3–22.8	15,012–18,182	1800–2249	1251–1452
SKJ (South Korea) *				
Concentration	119 ± 8.49	16,253 ± 483	1626 ± 70.0	1155 ± 73.1
Range	105–129	15,507–16,924	1556–1762	1066–1289
JA (Japan) *				
Concentration	64.8 ± 5.72	15,144 ± 756.4	1289 ± 48.2	962 ± 61.6
Range	58.6–74.2	14,034–16,451	1224–1373	863–1031
JM (Japan) *				
Concentration	123 ± 9.62	10,754 ± 703	2084 ± 215	1916 ± 142
Range	105–137.6	9816–11,989	1783–2464	1743–2189
JS (Japan) *				
Concentration	81.2 ± 10.8	18,576 ± 882	1444 ± 78.0	817 ± 40.4
Range	62.9–97.2	17,375–19,858	1332–1533	741–873

* CH—China (original); CH1—China (blended); I—India; SL—Sri Lanka; N—Nepal; SKJ—Jeoncha (South Korea); JA—Agari (Japan); JM—Matcha (Japan); JS—Sencha (Japan).

**Table 3:** The average content of trace elements in green tea samples according to their origin (including tea type or form).

Parameter	Trace Elements (mg/kg)				
	Mn	Zn	Cu	Fe	Cr
**CH (China)**					
Concentration	405 ± 15.0	35.6 ± 1.71	14.3 ± 0.11	79.1 ± 3.81	0.64 ± 0.07
Range	384–423	32.9–37.5	14.2–14.5	75.4–87.0	0.52–0.74
**CH1 (China)**					
Concentration	1461 ± 69.1	31.6 ± 4.47	17.4 ± 0.58	252 ± 18.9	1.16 ± 0.15
Range	1359–1547	26.9–39.9	16.2–17.9	231–290	0.96–1.34
**I (India)**					
Concentration	1213 ± 52.8	33.6 ± 7.19	13.1 ± 0.23	176 ± 16.4	1.03 ± 0.13
Range	1116–1266	25.6–46.1	12.9–13.5	146–195	0.88–1.26
**SL (Sri Lanka)**					
Concentration	551 ± 28.4	29.0 ± 4.46	11.4 ± 0.16	75.0 ± 4.77	0.37 ± 0.07
Range	518–592	24.5–37.0	11.1–11.7	69.0–84.3	0.28–0.46
**N (Nepal)**					
Concentration	300 ± 21.1	32.3 ± 2.86	12.2 ± 0.21	48.1 ± 4.90	0.67 ± 0.02
Range	270–340	27.5–36.1	11.9–12.4	40.7–56.7	0.64–0.68
**SKJ (South Korea)**					
Concentration	202 ± 6.90	37.6 ± 3.51	5.86 ± 0.11	51.0 ± 5.57	0.15 ± 0.02
Range	193–213	32.4–42.1	5.70–6.00	44.4–58.2	0.13–0.17
**JA (Japan)**					
Concentration	505 ± 12.8	36.2 ± 3.67	8.49 ± 0.18	109 ± 6.52	0.31 ± 0.03
Range	489–520	29.9–41.3	8.20–8.70	97.2–116	0.27–0.37
**JM (Japan)**					
Concentration	918 ± 40.6	22.9 ± 0.54	7.38 ± 0.13	154 ± 11.7	2.32 ± 0.18
Range	857–1004	21.9–23.5	7.20–7.50	138–172	2.09–2.55
**JS (Japan)**					
Concentration	480 ± 10.0	41.8 ± 4.08	8.81 ± 0.14	56.6 ± 4.15	0.15 ± 0.01
Range	461–489	36.1–47.4	8.50–8.90	52.1–64.9	0.14–0.16

CH—China (original); CH1—China (blended); I—India; SL—Sri Lanka; N—Nepal; SKJ—Jeoncha (South Korea); JA—Agari (Japan); JM—Matcha (Japan); JS—Sencha (Japan).

**Table 4:** The average content of toxic heavy metals in green tea samples according to their origin (including tea type or form).

Parameter	Heavy Metals		
	Pb (µg/kg)	Cd (µg/kg)	Ni (mg/kg)
**CH (China)**			
Concentration	9.37 ± 0.99	4.66 ± 0.61	5.09 ± 0.50
Range	8.30–11.23	3.98–5.72	4.32–5.80
**CH1 (China)**			
Concentration	238 ± 46.7	16.9 ± 2.09	7.92 ± 0.83
Range	168–311	12.6–19.2	6.89–9.10
**I (India)**			
Concentration	233 ± 40.7	11.7 ± 1.73	4.76 ± 0.59
Range	165–274	9.40–13.9	3.92–5.43
**SL (Sri Lanka)**			
Concentration	3.24 ± 0.51	3.02 ± 0.28	3.10 ± 0.47
Range	2.45–3.88	2.55–3.45	2.37–3.76
**N (Nepal)**			
Concentration	2.31 ± 0.14	4.51 ± 0.18	4.07 ± 0.24
Range	2.15–2.58	4.25–4.70	3.75–4.40
**SKJ (South Korea)**			
Concentration	31.8 ± 6.83	4.70 ± 0.25	18.0 ± 0.84
Range	20.2–41.3	4.25–4.95	17.0–19.2
**JA (Japan)**			
Concentration	20.5 ± 2.47	2.61 ± 0.30	5.28 ± 0.53
Range	17.4–24.6	2.23–3.13	4.66–5.97
**JM (Japan)**			
Concentration	29.8 ± 5.53	2.39 ± 0.17	2.13 ± 0.1
Range	22.0–37.0	2.13–2.60	1.97–2.28
**JS (Japan)**			
Concentration	<LOD	3.38 ± 0.22	4.75 ± 0.24
Range		3.10–3.80	4.53–5.19

CH—China (original); CH1—China (blended); I—India; SL—Sri Lanka; N—Nepal; SKJ—Jeoncha (South Korea); JA—Agari (Japan); JM—Matcha (Japan); JS—Sencha (Japan).

**Table 5:** Estimation of dietary exposure to bio- and toxic metals with green tea consumption.

Metal	Leaching Factor [%]	Appropriate Norm [mg]	Contribution to the Norm Regarding the Consumption of 2 g of Green Tea * [%]	
			Average	Range
Na	25.3	1500	0.002	0.0006–0.004
K	61.3	3500	0.50	0.33–0.70
Mg	35.1	400	0.31	0.23–0.37
Ca	17.6	1000	0.06	0.03–0.09
Mn	25.9	2.3	15.1	4.5–32.9
Zn	36.9	11	0.22	0.15–0.28
Cu	22.4	0.9	0.55	0.29–0.87
Fe	5.36	10	0.13	0.05–0.28
Cr	45.4	0.035	1.96	0.39–6.02
Pb	24.5	0.03	0.1	0.002–0.39
Cd	23.8	0.0214	0.01	0.005–0.04
Ni	51.3	0.3	2.09	0.73–6.16

* RDA or AI values for Na, K, Mg, Ca, Mn, Zn, Cu, Fe and Cr; TDI for Pb, Cd and Ni (60 kg adult men).

**Table 6:** Concentration of gallic acid and catechins in the green tea infusions.

Parameter	GA **	C **	EGC **	EGCG **	ECG **	EC **	Total
	**CH (China)**						
Mean (mg/100 mL)	6.34	24.9	76.7	107	86.2	41.7	343
SD	1.10	2.28	8.21	10.9	2.02	4.11	
Range	4.79–8.14	21.1–28.1	61.2–86.1	91.8–121	83.3–89.1	36.3–49.3	
	**CH1 (China)**						
Mean (mg/100 mL)	5.27	13.7	141	107	51.5	32.1	351
SD	0.51	0.92	11.4	3.14	3.60	3.69	
Range	4.16–5.81	11.9–14.9	128–160	103–113	44.8–56.2	26.4–37.8	
	**I (India)**						
Mean (mg/100 mL)	5.62	11.1	117	113	57.5	30.7	334
SD	0.16	0.87	4.34	6.88	1.98	3.22	
Range	5.35–5.85	9.87–12.2	109–124	103–123	53.1–59.4	25.8–35.8	
	**SL (Sri Lanka)**						
Mean (mg/100 mL)	10.6	19.8	157	123	59.0	35.1	404
SD	2.37	3.57	18.5	10.4	8.36	5.76	
Range	7.12–14.8	15.2–24.5	123–180	108–139	49.8–73.4	27.5–44.6	
	**N (Nepal)**						
Mean (mg/100 mL)	3.72	8.28	107	115	62.6	28.6	325
SD	0.95	1.77	8.21	8.54	7.40	4.46	
Range	2.81–5.35	6.15–11.5	96.5–119	102–129	52.3–74.5	22.6–34.8	
	**SKJ (South Korea)**						
Mean (mg/100 mL)	1.66	18.3	213	111	48.6	49.4	441
SD	0.08	1.72	28.1	8.10	4.81	3.48	
Range	1.54–1.83	15.8–21.3	175–249	101–123	43.3–56.2	44.2–54.5	
	**JA (Japan)**						
Mean (mg/100 mL)	2.46	17.8	180	110	50.9	41.8	403
SD	0.34	1.15	21.3	5.99	4.67	4.64	
Range	2.11–2.93	16.4–19.8	145–212	101–118	44.5–58.5	36.9–49.4	
	**JM (Japan)**						
Mean (mg/100 mL)	1.44	12.1	190	112	47.8	45.9	409
SD	0.03	1.27	24.8	7.30	4.74	2.48	
Range	1.39–1.48	10.1–13.9	158–231	99.4–122	41.1–54.8	41.8–50.8	
**JS (Japan)**							
Mean (mg/100 mL)	1.65	12.1	200	124	54.5	43.5	435
SD	0.07	0.67	12.0	3.43	1.09	0.96	
Range	1.55–1.78	11.3–13.3	187–218	118–129	52.3–55.7	41.2–44.5	
**Mean (mg/100 mL)**	**4.30**	**15.4**	**153**	**114**	**57.6**	**38.8**	**383**
SD	2.98	5.22	47.0	9.26	12.1	7.87	
Range	1.39–14.8	6.15–28.1	61.2–249	91.8–139	41.1–89.1	22.6–54.5	

* CH—China (original); CH1—China (blended); I—India; SL—Sri Lanka; N—Nepal; SKJ—Jeoncha (South Korea); JA—Agari (Japan); JM—Matcha (Japan); JS—Sencha (Japan); ** GA—gallic acid; C—catechin; EGC—epigallocatechin; EGCG—epigallocatechin-3-gallate; ECG—epicatechin gallate; EC—epicatechin.

**Table 7:** Antioxidant activity and TPC of the investigated green teas.

Tea	DPPH [%]	ABTS Trolox Equivalent [mM/L]	TPC [mg/100 mL]
CH (China) *	85.5 ± 5.13	12.4 ± 0.72	85.5 ± 3.14
CH1 (China) *	49.9 ± 2.07	7.12 ± 0.84	65.4 ± 2.52
I (India) *	68.0 ± 3.15	9.48 ± 0.76	63.6 ± 2.05
SL (Sri Lanka) *	85.6 ± 1.67	12.6 ± 1.08	97.3 ± 5.32
N (Nepal) *	74.3 ± 4.71	10.6 ± 1.28	85.9 ± 4.42
SKJ (South Korea) *	69.4 ± 5.34	9.72 ± 0.92	77.0 ± 4.05
JA (Japan) *	71.7 ± 5.44	10.0 ± 0.84	78.6 ± 5.00
JM (Japan) *	65.8 ± 0.7	9.12 ± 1.36	68.9 ± 6.67
JS (Japan) *	62.4 ± 2.24	8.72 ± 0.64	68.3 ± 2.69

* CH—China (original); CH1—China (blended); I—India; SL—Sri Lanka; N—Nepal; SKJ—Jeoncha (South Korea); JA—Agari (Japan); JM—Matcha (Japan); JS—Sencha (Japan).

**Table 8:** Selected validation parameters for metal analysis.

Parameter	Na	K	Ca	Mg	Mn	Zn	Cu	Fe	Cr	Ni	Cd	Pb
Reference value [mg/kg]	6300	10,260	3522	752.3	9.02	24.0	2.94	22.9	0.15	0.5	0.3	0.5
Determined value [mg/kg]	5712	10,820	3681	714.8	8.81	24.5	3.11	21.8	0.14	0.48	0.34	0.48
SD	211.2	678.4	224.5	62.1	0.44	1.14	0.12	1.05	0.01	0.04	0.03	0.02
RSD [%]	3.70	6.27	6.10	8.69	4.99	4.65	3.86	4.82	7.14	8.33	8.82	4.17
Recovery [%]	90.7	105.5	104.5	95.0	97.7	102.1	105.8	95.2	93.3	96.0	113.3	96.0
LOD [µg/kg]	84.0	56.0	185	34.0	184	38.0	189	172	0.72	0.31	0.09	1.32
LOQ [µg/kg]	286	242	612	116	634	141	661	558	2.78	1.27	0.34	4.84

## Supplementary Materials

The following are available online. Figure S1: Fragmentation spectra of all identified catechins and gallic acid obtained at the collision energy of 20 V in the negative ionization mode. Figure S2. Statistical significance of each parameter, separately. Table S1: Accurate mass measurements and identification data of catechins in the studied samples. Dataset S1: The raw data obtained from Principal Components Analysis.
