# Supplementary material for: Green Tea Quality Evaluation Based on Its Catechins and Metals Composition in Combination with Chemometric Analysis
Source: Molecules. 2018 Jul 11;23(7):1689. doi: 10.3390/molecules23071689 (PMC6100455; doi:10.3390/molecules23071689)
Supplement: Supplementary file 1 [file molecules-23-01689-s001.pdf]

SUPPLMENTARY MATERIAL

Green tea quality evaluation based on its catechins and metals composition in combination with chemometric analysis

Wojciech Koch, Wirginia Kukula-Koch, Łukasz Komsta, Zbigniew Marzec, Wojciech Szwerc and Kazimierz Głowniak

Table S1. Accurate mass measurements and identification data of catechins in the studied samples.

No	*Ion (+/-)*	*Rt (min)*	*Molecular formula*	*m/z experimental*	*m/z calculated*	*Delta (ppm)*	*DBE*	*MS/MS fragments*	*Proposed compound*
1	(-)	12.6	$C_{15}H_{14}O_6$	289.0717	289.0718	0.21	9.0	245, 203, 165	**Catechin**
2	(-)	11.4	$C_{15}H_{14}O_6$	289.0721	289.0718	-1.17	9.0	245, 203, 163	**Epicatechin**
3	(-)	10.2	$C_{15}H_{14}O_7$	305.0671	305.0667	-1.38	9.0	261, 219, 179, 167	**Epigallocatechin**
4	(-)	14.2	$C_{22}H_{18}O_{11}$	457.0790	457.0776	-2.98	14.0	305, 169	**Epigallocatechin gallate**
5	(-)	15.1	$C_{22}H_{18}O_{10}$	441.0841	441.0827	-3.12	14.0	289, 169	**Epicatechin gallate**
6	(-)	3.2	$C_9H_8O_4$	169.0150	169.0142	-4.43	5.0	125, 97	**Gallic acid**

Figure S1. Fragmentation spectra of all identified catechins and gallic acid obtained at the collision energy of 20V in the negative ionization mode.

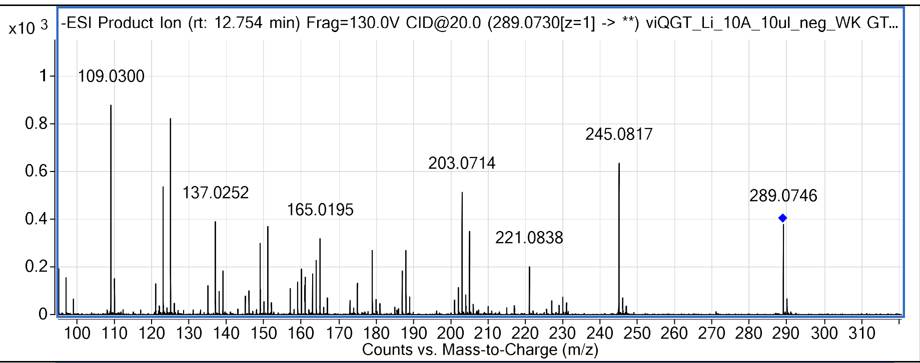

Catechin

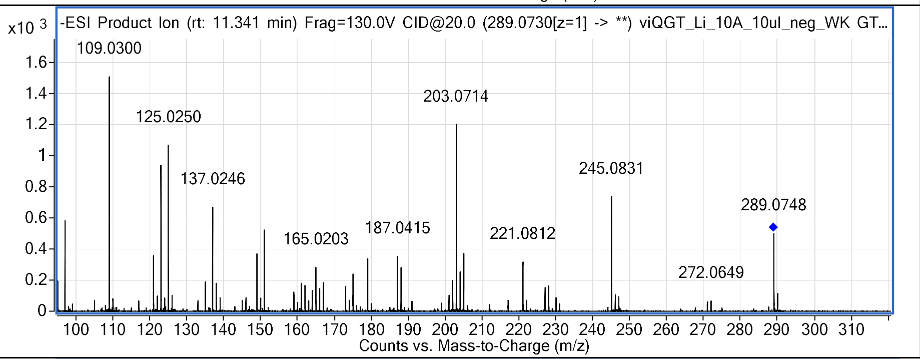

Epicatechin

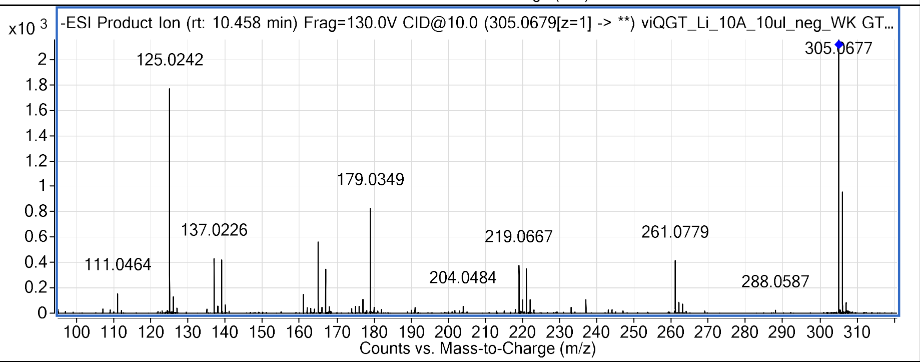

Epigallocatechin

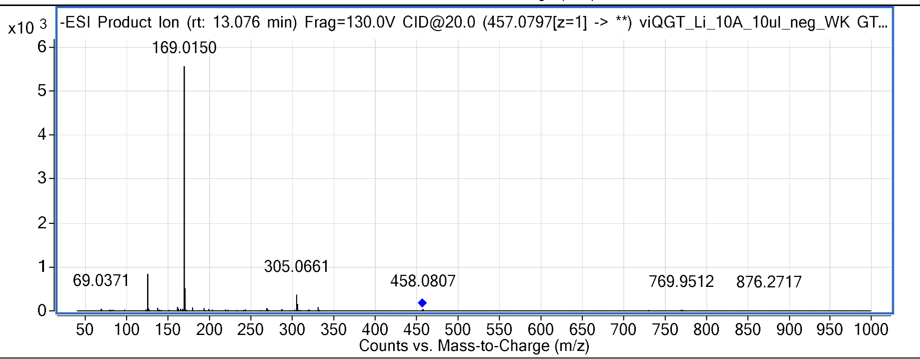

Epigallocatechin gallate

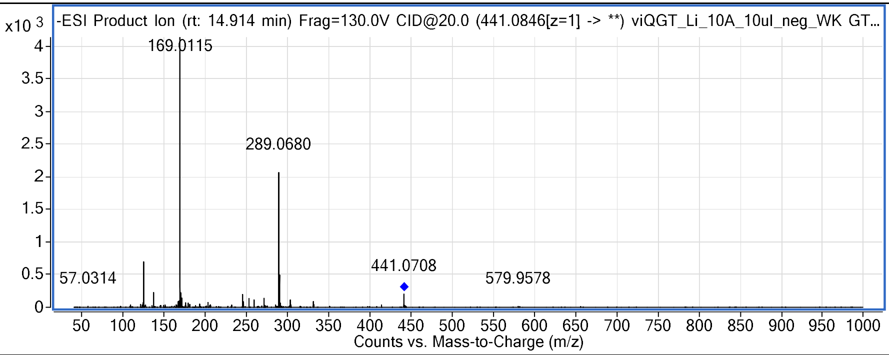

Epicatechin gallate

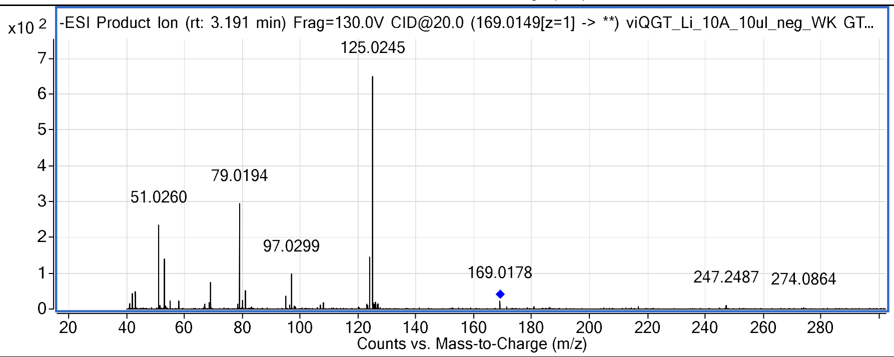

Gallic acid

PCA Summary and Scores

Dataset S1. The raw data obtained from Principal Components Analysis.

Table continues below

	PC1	PC2	PC3	PC4	PC5
**Standard deviation**	2.662	2.22	1.566	1.346	1.168
**Proportion of Variance**	0.3374	0.2348	0.1167	0.08627	0.06498
**Cumulative Proportion**	0.3374	0.5722	0.6889	0.7752	0.8401

Table continues below

	PC6	PC7	PC8	PC9	PC10
**Standard deviation**	0.9327	0.8342	0.6271	0.6098	0.5015
**Proportion of Variance**	0.04142	0.03314	0.01873	0.01771	0.01198
**Cumulative Proportion**	0.8816	0.9147	0.9334	0.9511	0.9631

Table continues below

	PC11	PC12	PC13	PC14	PC15
**Standard deviation**	0.4155	0.3822	0.3613	0.268	0.2565
**Proportion of Variance**	0.00822	0.00696	0.00622	0.00342	0.00313

**Cumulative Proportion**	0.9713	0.9783	0.9845	0.9879	0.991

Table continues below

	PC16	PC17	PC18	PC19	PC20
**Standard deviation**	0.2399	0.211	0.1876	0.1622	0.1262
**Proportion of Variance**	0.00274	0.00212	0.00168	0.00125	0.00076
**Cumulative Proportion**	0.9938	0.9959	0.9976	0.9988	0.9996

	PC21
**Standard deviation**	0.09146
**Proportion of Variance**	4e-04
**Cumulative Proportion**	1

Table continues below

PC1	PC2	PC3	PC4	PC5	PC6	PC7
-1.208	3.567	-0.4406	2.068	2.159	0.8809	-0.005901
-1.426	3.481	0.1338	1.997	0.7142	2.46	0.4478
-1.454	3.473	0.2349	1.572	1.61	1.068	-0.423
-1.116	3.285	-0.7634	1.497	2.767	0.5978	-0.3718
-1.419	3.058	-0.1135	1.895	1.734	0.5771	-0.7232
-1.399	3.517	-0.7839	1.176	0.6647	1.292	-0.05869
-1.395	3.803	-0.4604	1.329	0.7515	1.074	-0.04702
-1.198	3.474	0.1575	1.453	2.509	0.5237	0.03556
-1.585	3.205	-0.4896	1.49	0.4394	1.87	-0.0455
4.961	-1.108	-1.503	0.7523	-0.2467	0.4477	0.6496
5.818	-0.2463	-1.157	1.018	0.5381	-0.8099	0.2163
4.816	-0.9127	-1.696	0.2342	0.2332	-0.4638	-0.1152
4.415	-0.2519	-0.8184	-0.1008	-0.1015	-0.3296	0.4588
5.126	-0.3736	-0.7052	0.7357	-0.01584	-1.024	-0.155
5.669	-0.6722	-1.135	0.9683	-0.1955	-0.2981	0.3957
5.089	-1.099	-1.416	0.882	0.006128	0.3551	0.04222
5.261	-0.334	-1.648	0.36	0.03929	-0.3362	0.6179
5.103	-0.3943	-0.9838	0.5279	0.001402	-0.3324	0.8439
4.036	0.06305	-0.7865	-0.3261	0.1085	0.06531	-0.08024
3.383	-0.182	-1.348	-0.08731	-0.3863	0.2882	-0.5469
4.149	0.3915	-0.3539	-0.483	0.2489	-0.7719	-0.5958
2.917	0.6833	-1.487	-1.187	-0.9864	1.174	-0.04531
3.615	0.3662	-1.364	-0.8602	-0.7114	0.3999	-0.3125
3.931	0.1429	-1.518	-0.8177	-0.6489	0.7844	0.2089
3.729	0.2901	-0.7839	-0.305	0.00723	-0.06093	-0.2312
4.211	0.1179	-0.6834	-0.2574	0.5185	-0.399	-0.01262
3.65	0.1876	-1.336	-0.3645	-0.1508	0.6011	-0.61
-0.7981	3.742	0.3759	0.2708	-0.1586	-1.027	-0.7379
-0.4644	3.646	0.89	-0.3103	-0.5916	-1.226	0.4747
-0.6029	3.58	1.317	-0.01376	-3.61	-0.5962	0.09034
-0.9018	3.375	1.569	0.35	-3.422	-0.3616	0.5507
-1.169	3.083	0.972	0.5147	-2.894	-0.3146	-0.2675
-0.7759	2.769	0.7461	-0.2029	-3.312	0.5706	-0.1031

-0.7349	3.994	0.6206	1.098	-1.694	-0.1683	-0.4787
-0.469	3.535	1.092	0.6531	-2.29	-0.459	-0.08084
-0.726	2.939	0.7558	-0.4746	-1.876	-1.634	-0.3121
-1.207	1.376	-0.0236	-2.089	1.926	0.1665	1.686
-1.728	1.23	0.09168	-2.068	1.046	-0.8748	0.9329
-1.805	1.95	-0.7711	-2.597	0.692	-0.7782	0.3566
-1.331	1.459	0.03994	-3.28	0.6333	-0.5535	1.371
-0.981	0.9928	-0.2932	-2.727	0.2625	-0.3084	0.9029
-1.528	1.292	-0.157	-2.448	1.483	-1.524	0.6542
-1.61	0.9778	-0.6302	-2.258	1.812	-1.326	0.1352
-1.742	2.208	0.2927	-2.077	0.1821	0.517	1.669
-1.533	1.582	0.1207	-2.651	1.163	-1.516	0.6348
-2.639	-2.304	0.1249	2.429	0.63	-1.639	1.015
-2.459	-2.809	-1.111	1.54	0.1797	-1.071	0.5001
-2.573	-2.672	-0.6589	1.889	-0.8579	-0.03674	1.632
-2.834	-3.226	-0.4423	1.85	-1.452	-0.2199	1.471
-2.926	-2.215	-0.1052	2.372	-0.02292	-0.8741	0.7179
-2.443	-2.245	-0.641	2.525	0.3634	-0.8641	1.159
-2.378	-2.667	-0.2378	1.622	-0.7938	-0.3073	1.535
-2.452	-2.529	-0.5951	1.885	0.03575	-1.049	1.233
-2.842	-2.998	-0.8999	2.381	0.0174	-1.06	0.2374
-1.7	-1.956	-0.6263	0.07483	-0.2251	-0.03504	-1.774
-1.555	-0.1897	-0.2766	-0.1955	0.8031	-1.589	-1.632
-1.94	-1.584	-0.7439	-0.07026	-0.7471	0.5417	-1.718
-1.939	-1.05	-0.6401	-0.5017	-0.02944	-0.5549	-1.273
-1.978	-1.045	-0.4493	0.1572	0.4665	-1.354	-1.942
-1.654	-1.363	-0.6936	-0.1993	0.4713	-0.7208	-1.505
-1.561	-1.46	-0.5613	-0.3783	-0.4091	0.1367	-1.349
-1.918	-1.247	-0.6493	0.1739	-0.2501	0.1167	-1.648
-2.08	-0.6508	-0.3124	-0.4138	-0.008613	-1.322	-1.644
1.449	-1.539	3.831	-0.03676	0.6951	-0.08344	-0.03675
1.585	-2.116	4.036	-0.2707	-0.1091	1.209	0.2986
1.873	-1.975	3.071	-0.3527	1.013	0.4391	-0.6476
0.8353	-1.349	3.88	0.4334	1.038	-1.13	-0.8257
1.034	-2.129	4.353	0.3913	0.4804	0.2776	-0.55
1.876	-1.79	4.544	-0.3872	0.2099	1.228	0.9685

1.502	-1.847	4.303	-0.4946	0.191	0.6695	0.4962
1.098	-1.139	3.764	-0.0181	0.8057	-0.2438	-0.6657
1.524	-2.512	3.55	-0.2523	0.3248	0.844	-0.3834
-2.395	-2.411	-1.55	-1.392	-0.582	1.313	-0.1739
-2.196	-2.713	-1.419	-0.7367	-0.2175	1.566	-0.3535
-2.244	-2.351	-0.9756	-1.207	-0.5542	1.105	-0.3362
-2.076	-2.256	-0.8126	-1.275	-0.3986	1.275	0.1479
-2.256	-2.077	-1.021	-1.268	-0.05443	1.031	0.2425
-2.187	-1.924	-0.5968	-1.15	-0.1849	0.6769	0.01097
-2.231	-2.532	-1.143	-1.29	-0.4351	0.9092	-0.5241
-2.747	-2.267	-1.223	-1.215	-0.7955	1.196	-0.2139
-2.15	-2.126	-0.8322	-1.475	-0.5571	1.401	0.5153

Table continues below

PC8	PC9	PC10	PC11	PC12	PC13	PC14
-0.07301	-0.4367	-0.2642	0.5096	-0.2555	0.2562	-0.1207
0.4684	-0.2946	0.9281	0.2465	0.1711	-0.07152	0.2705
0.2197	0.6154	0.664	-0.2237	0.1142	0.4312	0.1739
-0.3738	0.06452	-0.6483	0.02645	-0.5707	0.05456	-0.08758
0.6337	-0.08475	0.5282	-0.09988	0.0341	0.1518	0.1372
0.3409	-0.4098	0.3699	-0.3363	0.4911	-0.5861	-0.2674
0.3255	0.2327	0.1438	-0.2828	0.3803	0.02869	-0.4312
-0.6787	0.004656	-0.6727	0.1896	-0.4085	0.1274	-0.2739
-0.2165	0.3182	0.4933	-0.1734	0.02717	-0.5231	-0.1307
0.4477	-0.295	-0.5926	1.182	0.4392	-0.4422	-0.531
0.5695	-0.4013	0.4324	-0.155	0.4581	0.3847	0.7249
0.9326	-0.1025	-0.2503	-0.2865	0.4748	-0.08516	-0.5681
1.017	-1.004	0.4319	-0.4762	-0.5125	-0.7835	0.2143
0.8617	-0.4408	0.01345	0.007577	0.327	0.00624	-0.01169
0.05145	-0.7453	-0.2202	0.05212	0.3713	0.1716	-0.1218
-0.1097	-0.754	-0.2714	0.8172	-0.1574	-0.003711	0.09778
0.6882	-0.4449	-0.1205	-0.3691	0.0535	-0.4438	-0.1269
0.3854	-1.138	0.1975	-0.0509	0.1944	0.05809	-0.09903
-1.206	-0.1164	0.7606	-0.4549	-0.1955	0.3034	0.08448
0.4311	0.8988	0.4575	0.1391	-0.423	0.4918	0.05052
-1.217	0.7163	0.7552	-0.05105	-0.3965	0.6244	0.1927
0.3722	1.962	-0.905	0.3445	-0.2478	-0.53	-0.2329

-0.3337	1.305	0.5085	-0.4862	-0.1186	0.04461	0.1125
-0.6485	0.8228	-0.2835	-0.2462	-0.008126	-0.01625	-0.04163
-0.9812	0.08467	-0.02406	-0.1498	-0.3934	0.1116	-0.1707
-1.217	0.03783	0.424	-0.3104	-0.7016	0.4604	0.2473
-0.576	1.264	-0.4327	0.3649	0.1878	0.1357	0.04321
0.7415	0.6805	-0.7653	-0.3167	-0.2912	-0.372	0.5157
-0.1865	-0.5104	-0.7354	-0.5572	-0.4669	-0.3996	-0.2302
-0.6279	-0.5635	0.02822	0.37	-0.08094	0.7174	-0.784
-0.1777	-0.9915	0.2974	0.2148	0.09479	-0.359	0.1238
0.2185	-0.7286	0.6247	0.5719	-0.5029	0.4269	0.01415
0.6638	1.463	-0.2088	0.1851	0.6321	0.611	0.2399
-0.1193	0.1127	-1.58	-0.4883	0.3723	0.1726	0.5328
-0.5745	-0.8697	-0.4771	-0.2462	-0.3049	-0.569	0.5185
0.451	0.1431	0.01044	-0.1372	-0.8924	0.08573	-0.3046
-0.312	-0.6609	-0.8451	0.06329	0.02078	0.5737	0.3888
0.272	-0.595	-0.02699	0.6693	0.4297	0.43	0.09076
0.9172	0.8938	0.2663	-0.1265	0.1613	-0.4794	-0.1151
-0.7268	0.4436	0.6528	-0.5361	0.2123	-0.4173	-0.03364
-1.051	0.2094	0.02632	0.6906	0.5858	-0.2788	0.1446
-0.2486	0.4419	-0.2188	0.7842	-0.3914	-0.2832	-0.1223
0.153	0.01983	-0.4679	0.4359	-0.2296	0.1131	-0.04621
0.04484	-0.5021	0.5869	0.01422	0.9529	0.3237	0.0602
0.7574	0.3232	0.3595	-0.38	0.5556	0.3779	-0.09137
0.1914	0.01145	0.5982	0.639	-0.6525	0.3096	-0.07231
0.949	0.8873	-0.3265	0.01931	0.01972	0.4092	0.399
-0.869	0.6697	-0.6688	-0.7709	0.4788	-0.4068	-0.2816
-0.1376	0.6644	0.1587	-0.02474	0.2314	0.02138	0.03686
-0.7374	0.4914	0.4168	-0.2779	-0.2863	0.2054	-0.412
-0.9696	-0.1981	-0.4245	-0.4469	0.4405	-0.2	0.07189
-0.3242	0.2615	0.9797	0.1689	0.2173	-0.09438	0.02954
0.1751	0.8985	-0.0304	0.3084	-0.3171	-0.2725	0.05203
0.3345	0.2004	-0.4945	0.0444	-0.1734	0.1309	0.4273
-0.5238	-0.5505	-0.1726	0.8941	0.6578	0.3654	0.2188
-0.7738	-0.2249	0.4789	-0.1428	0.2417	-0.3157	-0.2807
-0.3479	0.2606	0.263	0.5051	0.3396	-0.5073	0.2677
0.8257	-0.1162	0.4079	-0.5346	0.05974	-0.1959	-0.1726

0.05583	-0.3497	0.01016	-0.2151	0.3916	-0.08124	0.1568
-0.07345	-0.9207	-0.5641	-0.7399	0.4073	-0.1322	0.06632
-0.6835	-0.02542	0.3653	0.4738	0.07724	-0.7578	0.2425
-0.4118	0.105	0.6054	0.829	0.1736	-0.1665	-0.2277
-0.2449	-0.01396	0.5207	-0.03187	0.2499	-0.2928	-0.2735
-0.4454	-0.2941	-0.1618	0.5006	0.005884	-0.4344	0.01727
-0.7852	-0.09914	-0.18	-0.1639	0.6204	0.1921	0.03551
-1.272	-0.02707	-0.43	-0.2969	0.1844	-0.2031	0.2089
0.8741	0.02499	-0.5421	0.1964	-0.2229	0.09388	-0.1773
0.4707	0.05451	-0.3569	-0.5132	0.3398	0.7907	-0.5985
0.6044	0.3139	0.3974	0.4572	-0.5774	-0.8192	0.2275
0.6254	0.4405	-0.03393	0.1131	-0.1112	-0.03053	0.1451
1.087	0.5538	0.5602	0.1215	0.05588	-0.09196	0.1202
-0.4028	-0.002958	-0.06084	-0.5936	-0.09436	0.09563	-0.1862
0.6074	-0.4913	-0.7547	0.2418	-0.1097	0.1083	0.2458
-0.3399	-0.2302	-0.9177	0.1368	-0.2545	0.1173	-0.2704
0.5124	-0.1298	0.1778	-0.208	-0.1816	0.2586	-0.1232
-0.4814	-0.4445	0.1823	-0.3832	-0.5009	0.105	0.1172
-0.000576	-0.9752	-0.3093	0.08595	-0.5074	0.1904	0.04467
0.2858	-0.6398	0.3307	-0.4579	-0.6306	-0.205	0.008531
0.6201	-0.07855	-0.05079	-0.3739	-0.2702	0.4525	0.2223
1.18	-0.1194	-0.1972	-0.2567	-0.2292	0.2107	-0.3479
0.118	-0.8736	0.2741	-0.2418	-0.2659	0.1197	0.0262

PC15	PC16	PC17	PC18	PC19	PC20	PC21
-0.08901	-0.3909	0.1151	-0.07955	-0.03116	-0.1228	-0.06373
-0.01418	0.4132	0.05759	0.1588	-0.08937	-0.1944	0.04602
0.3533	0.2374	-0.4119	0.05118	0.1848	0.07995	-0.0261
-0.06858	-0.3801	-0.111	0.0477	0.07232	0.115	0.006195
-0.3442	-0.07544	-0.0949	-0.2023	0.09652	0.1655	0.05539
-0.4015	-0.104	0.2412	-0.1882	0.00832	0.06566	0.1479
-0.1837	-0.3173	0.04666	-0.0899	-0.2093	0.06785	0.03186
0.6475	0.1455	-0.1826	0.1946	-0.1781	0.325	-0.0108
0.3938	0.02108	0.0379	-0.1264	0.1902	-0.09867	-0.01547
-0.1452	0.02574	-0.567	-0.4155	-0.04721	-0.01461	-0.3427
0.0135	-0.04435	0.09204	-0.04228	0.004947	0.1574	-0.03373
0.07155	-0.0608	-0.0329	-0.1196	0.35	0.02678	0.1792

-0.3441	-0.2029	-0.1642	0.2049	0.3234	0.2744	-0.1748
0.2103	0.05286	-0.4526	0.5472	-0.3521	-0.17	0.2886
0.5906	0.2	0.0503	0.04168	-0.4123	0.01973	0.06937
-0.2239	-0.08943	0.3134	0.008389	0.1526	0.1383	-0.03623
-0.03003	-0.1148	0.04532	0.2396	-0.2667	0.09097	-0.05734
0.4644	-0.1038	-0.1635	-0.2367	0.2183	-0.2191	0.1155
-0.07664	-0.008528	-0.0651	-0.1874	0.03351	-0.2053	-0.05467
0.07615	0.0412	0.5191	0.09946	0.01219	-0.04048	0.008012
0.007557	0.1866	0.162	-0.5256	-0.1138	0.1446	0.0604
0.04399	0.09344	0.02454	0.3434	0.1578	-0.1734	0.02168
-0.02732	0.1423	0.4505	0.3079	0.1147	0.2471	-0.09547
-0.3926	0.3125	0.151	-0.5727	-0.1961	-0.1443	0.01272
-0.3204	0.1899	-0.3491	-0.0155	0.1787	-0.2871	-0.1029
0.1438	-0.1575	-0.2091	0.1616	-0.2661	-0.09873	-0.1029
-0.002867	-0.07565	0.5261	0.1878	0.1921	0.1401	0.1556
-0.2647	0.6035	-0.2615	-0.06022	-0.05687	-0.04925	-0.001423
-0.03896	0.8098	0.03998	0.05308	0.4288	-0.07203	0.07307
-0.3019	-0.5598	0.08222	0.2979	-0.09938	0.1297	0.01774
0.434	0.3333	0.1819	-0.1538	0.08743	0.03939	0.07029
-0.07615	-0.2611	0.1077	-0.01593	0.09034	-0.09383	-0.0568
0.01399	0.1169	-0.5519	0.1389	0.02771	0.1222	-0.0244
-0.1078	-0.04447	0.03606	0.03389	-0.2052	-0.1471	-0.1868
-0.08007	-0.357	0.1179	-0.2181	-0.2362	-0.04571	0.1358
0.09816	-0.248	-0.2043	-0.1087	0.162	0.09663	0.003184
-0.07535	0.02807	0.1287	0.4048	0.04093	-0.1757	-0.08393
0.154	0.3032	0.1717	-0.04425	0.2004	-0.02205	0.01307
0.009679	-0.6339	0.2305	-0.1479	-0.1846	-0.215	-0.03705
0.3713	-0.0836	-0.2335	-0.08821	0.01534	0.1064	-0.01757
-0.3386	-0.177	-0.2145	-0.1483	-0.04556	0.1292	0.1404
0.2441	-0.0344	-0.03577	-0.114	-0.08085	0.0572	0.1101
0.02065	-0.09751	0.06329	-0.1093	0.1907	-0.1235	0.05755
-0.04963	0.01045	0.3624	0.1556	-0.1186	-0.147	-0.1621
-0.1163	0.08865	-0.1527	0.005171	-0.0664	0.08113	-0.01296
-0.2937	0.2965	0.01774	0.1218	-0.041	-0.08786	0.06582
-0.2289	-0.3647	-0.1277	0.01693	0.1172	-0.04502	0.05549
0.09115	0.0146	0.1377	0.1033	0.0005425	0.07176	-0.03084

0.5071	-0.1904	-0.1299	-0.07394	0.2029	0.04561	-0.07713
0.2636	0.2288	0.02889	0.04249	-0.1283	-0.135	-0.02432
-0.1747	0.1081	0.2449	-0.005784	0.004333	-0.005285	-0.06107
-0.5684	0.1156	-0.1649	0.02201	0.104	0.02299	0.08866
-0.3668	-0.005542	0.03421	0.05683	-0.148	0.1033	0.02271
0.4092	-0.4037	0.1429	-0.2511	0.08813	-0.1012	0.02494
-0.09018	0.1345	-0.06561	0.1363	0.127	0.1069	0.0801
-0.2033	0.0789	-0.03994	0.3417	6.91e-05	-0.01034	-0.1336
0.35	-0.01214	-0.04844	-0.07398	-0.0229	-0.1204	-0.00462
-0.07539	-0.1383	0.1433	0.1338	-0.05425	-0.1598	-0.0294
-0.0003466	0.003575	0.1137	-0.1749	0.1129	0.06731	-0.126
-0.1358	0.004602	0.1804	0.1246	-0.01304	0.01845	0.0444
0.4242	-0.1128	-0.06503	0.06254	-0.05104	-0.05522	0.0639
-0.1066	0.1128	0.0347	0.209	0.06262	-0.2071	-0.07565
0.3839	0.06923	0.01485	0.03149	-0.1411	0.1646	-0.1348
-0.328	0.2749	-0.01769	0.06908	-0.1096	0.1421	-0.007173
-0.1057	0.02894	-0.04196	-0.1916	0.05759	0.1278	0.02242
-0.1879	-0.4341	-0.3431	0.1411	0.1636	-0.1206	0.1101
0.08871	0.3088	0.3164	-0.2612	-0.1706	0.0417	-0.04964
0.1883	-0.005725	0.09259	-0.06379	0.1351	-0.08599	-0.03079
0.1506	-0.1479	0.2034	0.1719	-0.05275	-0.04138	-0.03354
0.2248	-0.0976	0.03585	-0.009834	-0.1447	0.1421	-0.03062
-0.2877	0.06507	-0.01801	-0.04435	-0.05199	-0.1071	-0.04396
0.004411	-0.3413	-0.0314	0.03393	0.2152	-0.1279	0.07976
0.325	-0.08489	0.09651	-0.0516	0.1441	0.07363	-0.051
-0.3302	0.1569	-0.05986	-0.06465	-0.2624	0.04916	0.05851
-0.3529	0.1854	-0.1964	-0.06276	-0.1587	0.03637	0.1139
0.1345	-0.06446	-0.2466	0.000851	-0.02571	0.04672	-0.03479
0.06192	0.1496	0.08871	0.0329	0.1088	0.03769	-0.03454
-0.04095	0.119	-0.04703	-0.04257	-0.2533	0.06859	0.04304
0.02167	-0.1902	-0.3168	-0.1268	-0.05514	0.0517	0.01131
0.09085	0.2777	0.1335	-0.1795	-0.1482	-0.02884	0.003416
-0.09118	0.1269	0.003111	0.1528	0.1105	0.06066	0.005165

PCA loadings

Table continues below

	PC1	PC2	PC3	PC4	PC5	PC6
**C**	-0.1225	0.1802	-0.02789	0.572	-0.06354	0.153
**EC**	-0.1722	-0.2174	0.2006	0.3945	-0.0381	0.2851
**EGC**	-0.0992	-0.3411	0.1276	0.08966	-0.3458	-0.1236
**ECG**	-0.06523	0.3303	-0.1095	0.1039	0.3518	0.3855
**EGCG**	-0.0861	0.01024	0.03225	-0.2691	-0.6305	0.4338
**GA**	0.08276	0.3676	-0.04787	0.09691	-0.3891	-0.0497
**K**	-0.2948	-0.03944	-0.239	-0.1335	0.1446	0.07328
**Ca**	0.279	0.2148	0.1233	0.1046	-0.2552	-0.1036
**Mg**	0.02214	0.2608	0.367	0.07261	0.1823	0.1029
**Na**	-0.009291	-0.337	0.2523	0.3345	-0.02702	0.111
**Fe**	0.352	-0.06376	-0.002897	0.08125	0.0157	-0.0009811
**Mn**	0.3647	-0.04493	-0.001845	-0.01035	-0.05789	0.07126
**Cu**	0.2459	0.2577	-0.1899	0.2374	-0.06207	0.1352
**Zn**	-0.1477	-0.0941	-0.4649	0.02793	-0.03017	0.3501
**Pb**	0.3356	-0.05005	-0.2194	0.06675	-0.01294	-0.07085
**Cd**	0.3145	-0.01163	-0.2916	0.08309	0.0297	-0.07283
**Ni**	-0.07371	-0.1924	-0.2333	0.4279	-0.01676	-0.314
**Cr**	0.2335	-0.04888	0.4393	-0.05398	0.2147	0.1375
**TPC**	-0.2028	0.3063	0.09774	0.0601	-0.1431	-0.3316
**DPPH**	-0.2001	0.3104	0.1196	0.076	-0.06573	0.01159
**ABTS**	-0.2643	0.1293	-0.006854	0.002457	0.03929	-0.3435

Table continues below

	PC7	PC8	PC9	PC10	PC11	PC12
**C**	-0.2614	0.01861	-0.2109	0.1018	-0.04301	0.1451
**EC**	-0.07752	-0.01537	-0.1606	0.2669	0.4172	-0.07096
**EGC**	-0.09184	0.06957	-0.1666	-0.479	-0.4096	0.08276
**ECG**	0.0189	0.01561	0.03028	-0.07068	-0.5795	0.2102
**EGCG**	0.2534	0.1778	0.1008	0.362	-0.1373	0.2286
**GA**	-0.01315	-0.03515	-0.1355	-0.08567	0.07963	-0.1916
**K**	0.2448	0.09521	-0.4467	0.03868	-0.07302	-0.4699
**Ca**	0.09222	0.07795	0.1738	-0.2315	-0.000938	-0.3244
**Mg**	0.5272	0.3362	-0.01864	-0.2003	0.1717	-0.02493
**Na**	0.07407	0.09227	0.1611	-0.06976	-0.2531	-0.2763
**Fe**	-0.2114	0.1505	-0.1312	0.03705	0.0185	0.247

**Mn**	-0.1528	0.1108	-0.1115	-0.001983	-0.01682	-0.1454
**Cu**	-0.004337	0.1253	-0.08248	-0.003911	-0.04575	-0.1902
**Zn**	-0.08587	0.2028	0.3378	-0.5288	0.3854	0.09725
**Pb**	0.01122	-0.07694	0.1651	0.1799	-0.07244	-0.03633
**Cd**	0.2209	0.09434	-0.09755	0.1111	0.04522	0.1419
**Ni**	0.5152	-0.05419	0.2605	0.1302	-0.0648	0.169
**Cr**	-0.004708	0.07559	0.1432	-0.03426	0.08365	0.191
**TPC**	0.06599	-0.03327	-0.2615	-0.1759	0.1426	0.4177
**DPPH**	-0.1076	-0.4417	0.4438	0.04685	-0.02398	-0.1857
**ABTS**	-0.3079	0.7165	0.2746	0.2618	-0.07569	-0.0877

Table continues below

	PC13	PC14	PC15	PC16	PC17	PC18
**C**	-0.4978	0.2953	-0.01989	0.002446	0.1933	-0.259
**EC**	0.5425	0.012	0.1397	0.07777	-0.04616	0.1062
**EGC**	0.2018	0.1478	0.4153	-0.1319	-0.002578	-0.1124
**ECG**	0.3547	-0.03675	-0.09486	0.02369	0.01429	0.1416
**EGCG**	-0.1085	-0.04927	0.03444	0.07668	0.03549	-0.00485
**GA**	0.2276	-0.1173	-0.2795	-0.6241	0.1449	0.09083
**K**	-0.1688	-0.254	0.2392	-0.05486	0.3079	-0.007512
**Ca**	0.003448	0.1751	-0.08237	0.3261	0.003688	0.008235
**Mg**	-0.06997	0.2823	0.2295	-0.006642	0.04278	0.1697
**Na**	-0.1318	-0.4398	-0.3205	0.1845	0.02819	0.05555
**Fe**	-0.2533	-0.2115	0.272	-0.1428	-0.02364	0.6605
**Mn**	-0.03211	-0.1454	0.03667	0.1545	0.05949	0.02855
**Cu**	0.04323	-0.1873	0.1129	0.04606	-0.3506	-0.1655
**Zn**	-0.0409	-0.1051	0.0004101	0.005573	0.121	0.001402
**Pb**	0.2412	0.1578	0.1518	0.1812	0.714	0.01654
**Cd**	0.106	-0.1644	0.2684	0.04308	-0.2613	-0.4103
**Ni**	-0.05308	-0.0389	-0.04774	-0.2357	-0.01742	0.1184
**Cr**	-0.03	-0.3037	0.04804	-0.3564	0.2819	-0.4451
**TPC**	0.09758	-0.437	-0.06046	0.4141	0.1988	-0.001979
**DPPH**	-0.1065	-0.2336	0.5497	-0.01277	-0.03683	0.04157
**ABTS**	0.1193	-0.04281	0.0795	-0.04321	0.01657	-0.04151

	PC19	PC20	PC21
**C**	-0.04701	-0.08516	-0.03717
**EC**	0.1239	-0.1123	-0.02736
**EGC**	0.01303	0.06506	-0.00411

**ECG**	0.0739	-0.1814	-0.09797
**EGCG**	0.05058	0.00443	0.00146
**GA**	-0.2322	-0.07448	-0.01983
**K**	0.2067	-0.1509	0.02553
**Ca**	0.4325	-0.4821	0.08442
**Mg**	-0.295	0.169	-0.05111
**Na**	-0.3652	0.0007018	0.1666
**Fe**	0.08083	-0.1646	0.2104
**Mn**	-0.02782	0.1237	-0.848
**Cu**	0.2599	0.6007	0.2594
**Zn**	-0.01152	-0.02222	-0.02562
**Pb**	-0.1419	0.2097	0.2043
**Cd**	-0.3969	-0.4387	0.04771
**Ni**	0.3058	0.05551	-0.2492
**Cr**	0.3289	-0.04249	0.03531
**TPC**	0.02707	0.07662	-0.01014
**DPPH**	-0.1219	-0.04231	-0.1005
**ABTS**	-0.01532	-0.03106	-0.02899

Figure S2. Statistical significance of each parameter, separately.

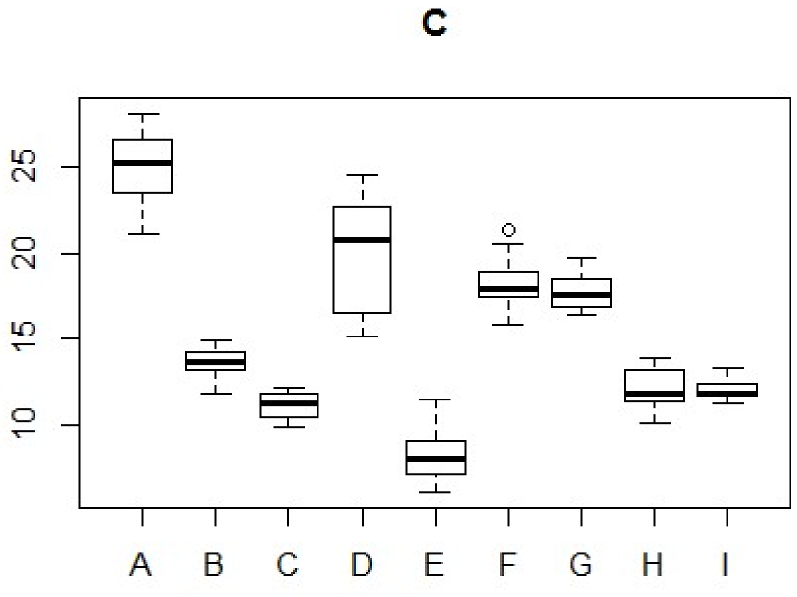

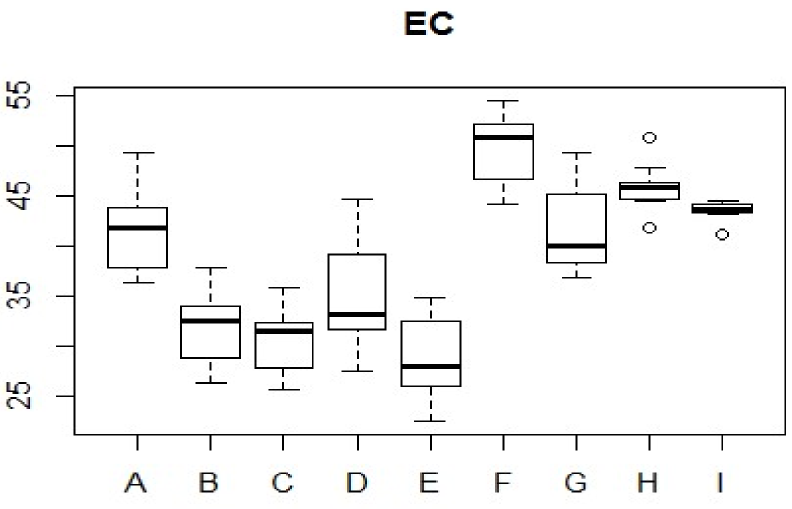
EC
55
45
35
25
A
B
C
D
E
F
G
H
I

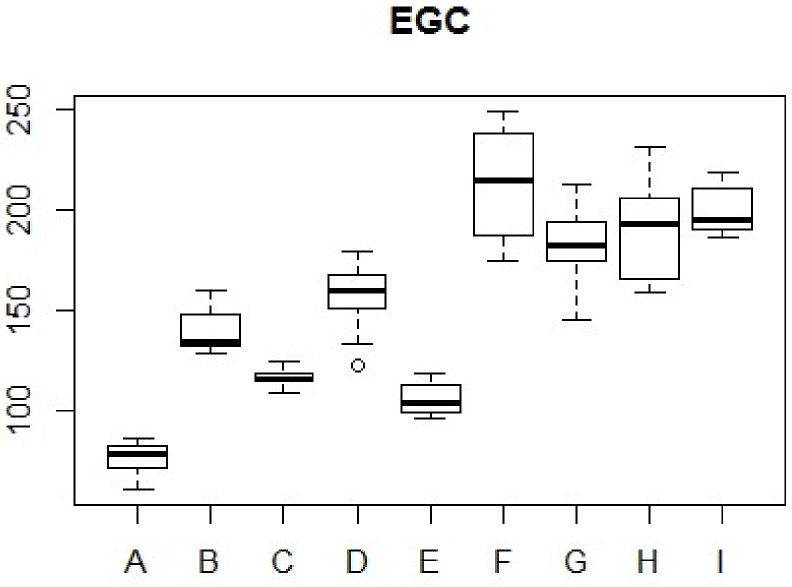
EGC
250
200
150
100
A
B
C
D
E
F
G
H
I

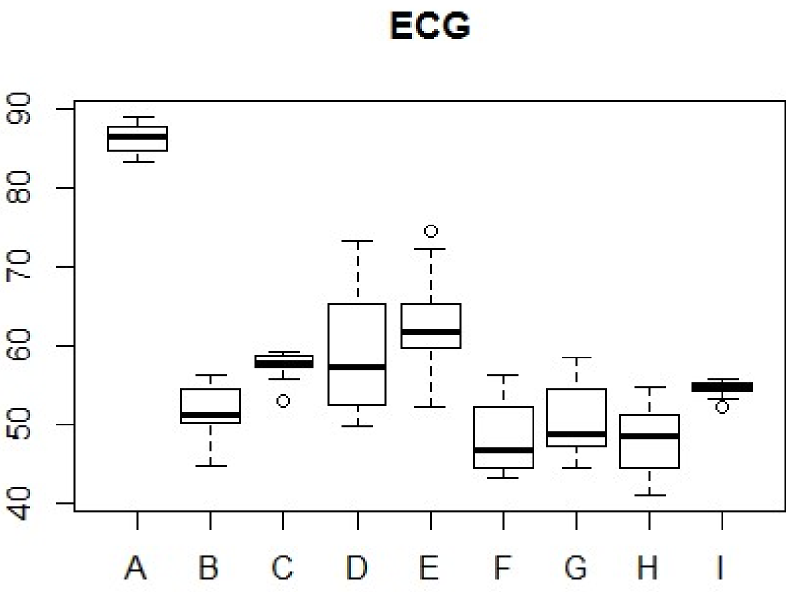
ECG
90
80
70
60
50
40
A
B
C
D
E
F
G
H
I

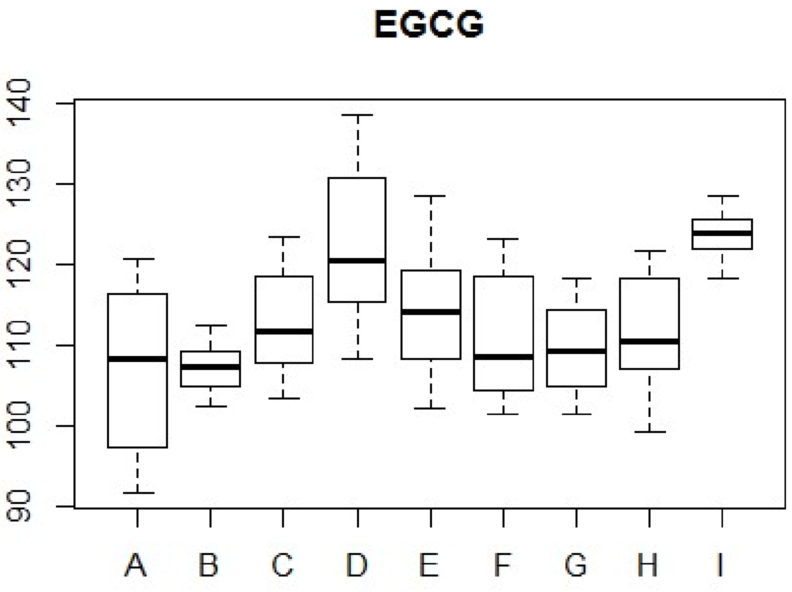
EGCG
140
130
120
110
100
90
A
B
C
D
E
F
G
H
I

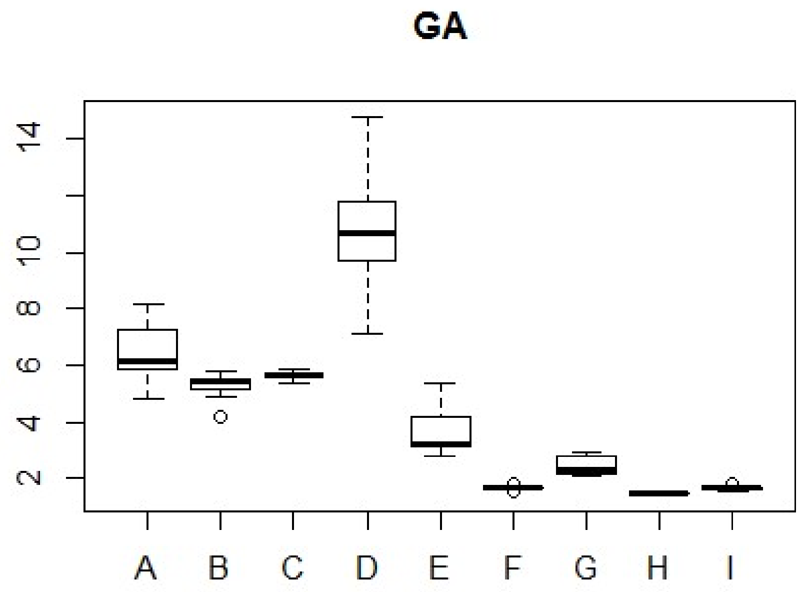
GA
14
10
8
6
4
2
A
B
C
D
E
F
G
H
I

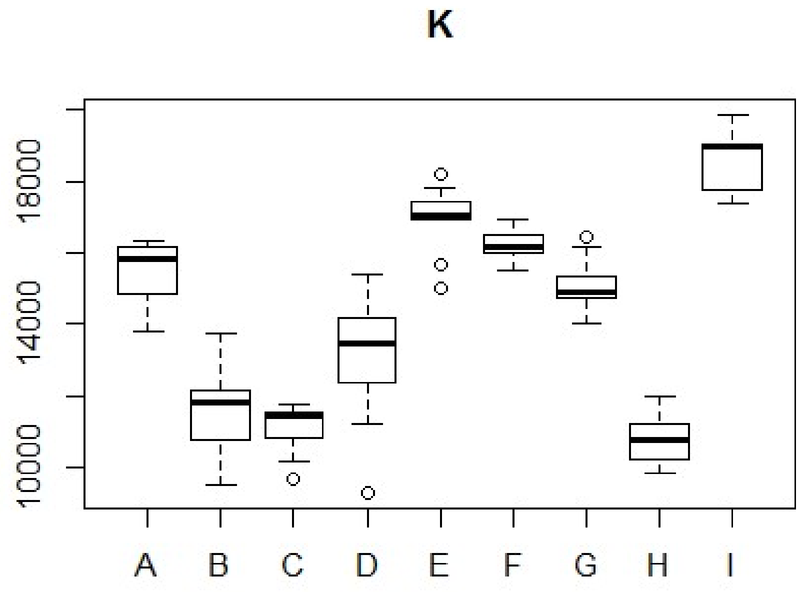
K
18000
14000
10000
A
B
C
D
E
F
G
H
I

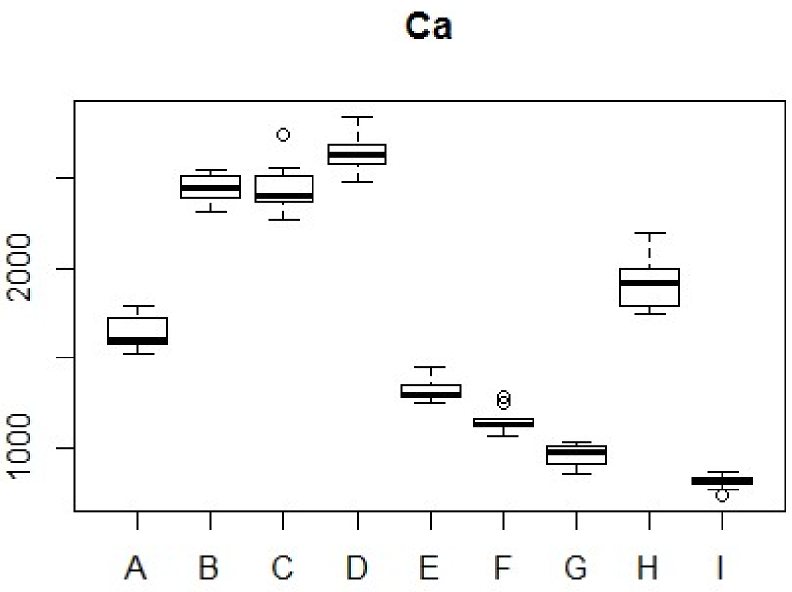
Ca
1000
2000
A
B
C
D
E
F
G
H
I

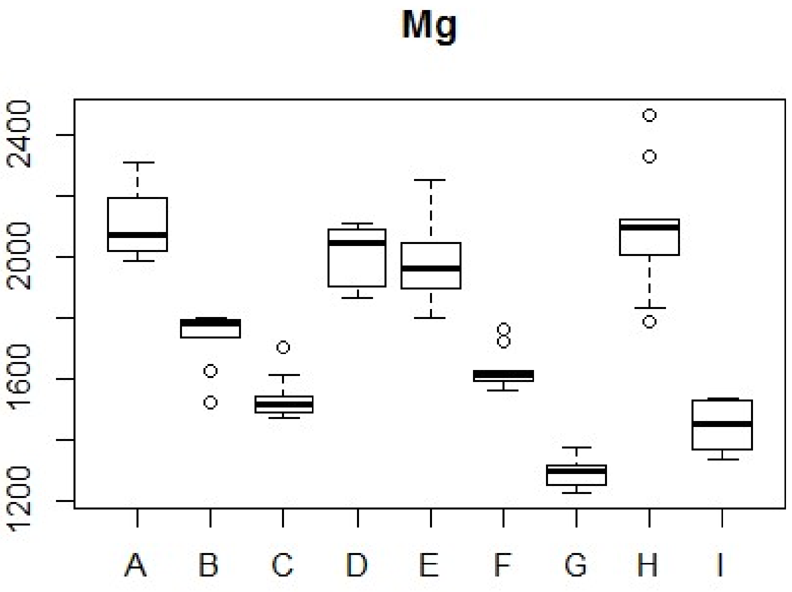
Mg
1200
1600
2000
2400
A
B
C
D
E
F
G
H
I

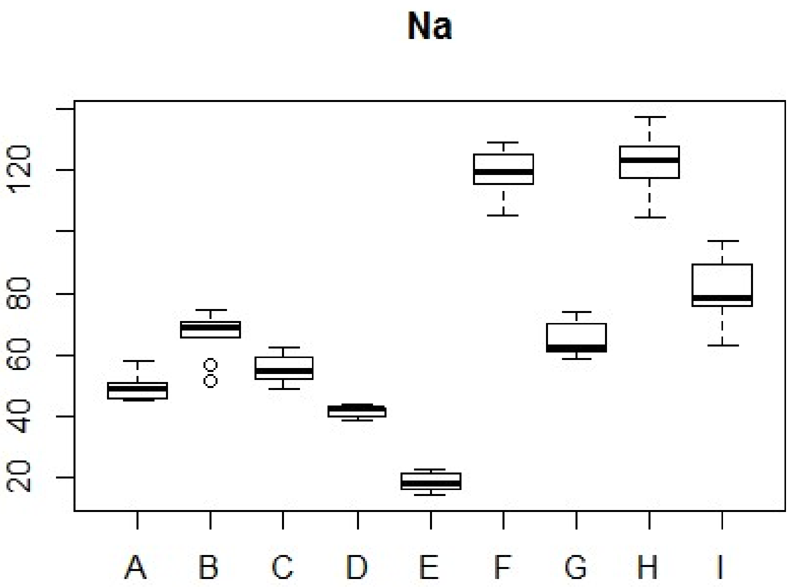
Na
20
40
60
80
120
A
B
C
D
E
F
G
H
I

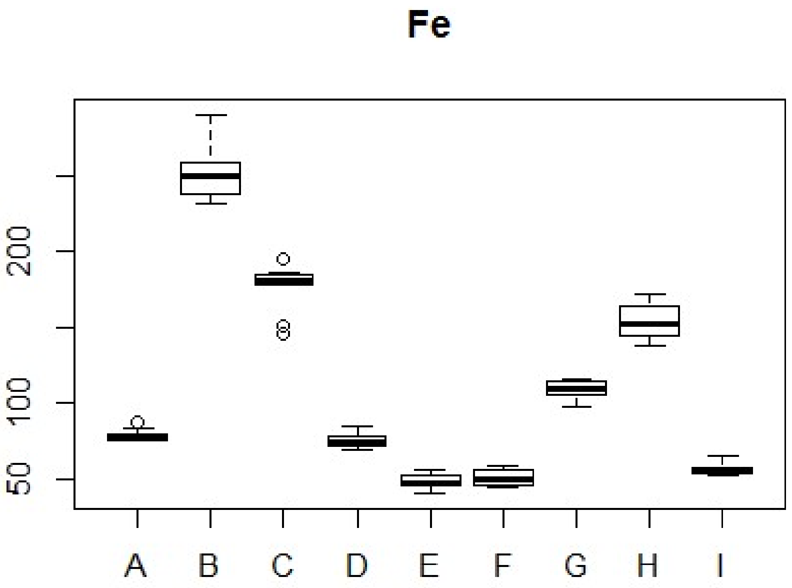
Fe
50
100
200
A
B
C
D
E
F
G
H
I

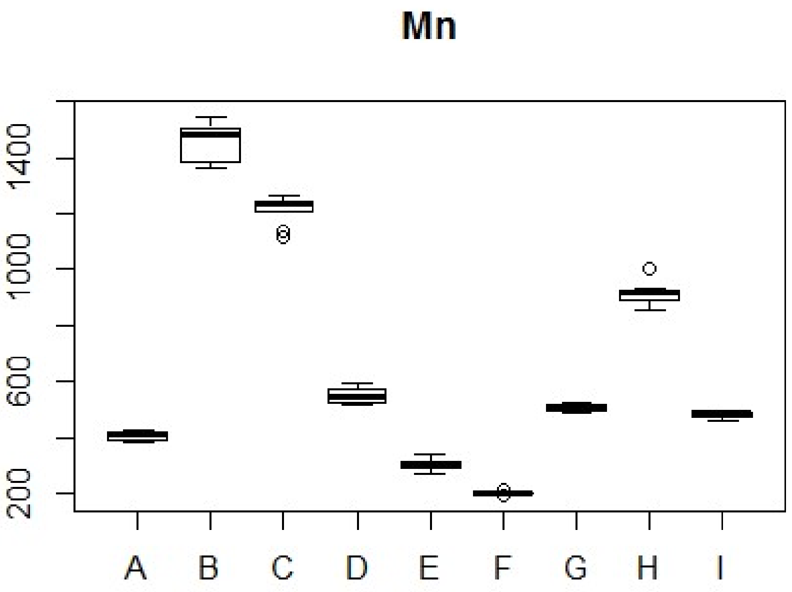

Mn
1400
1000
600
200
A
B
C
D
E
F
G
H
I

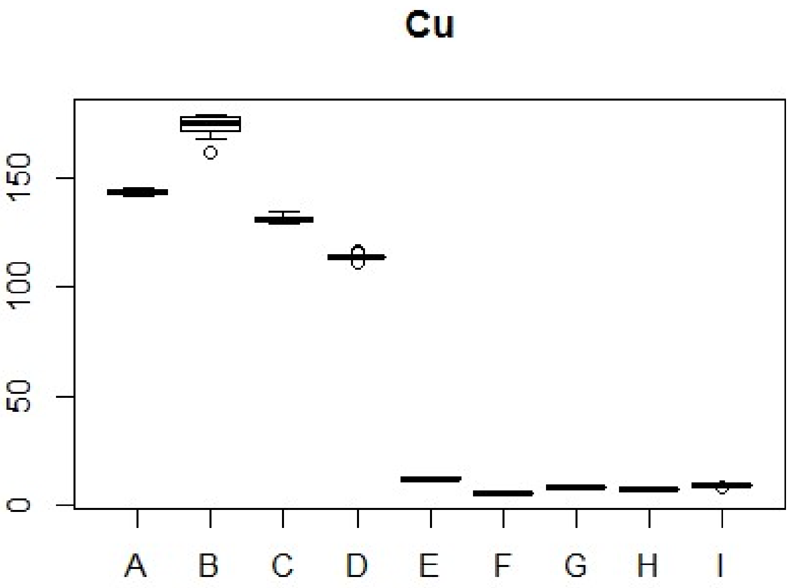

Cu
150
100
50
0
A
B
C
D
E
F
G
H
I

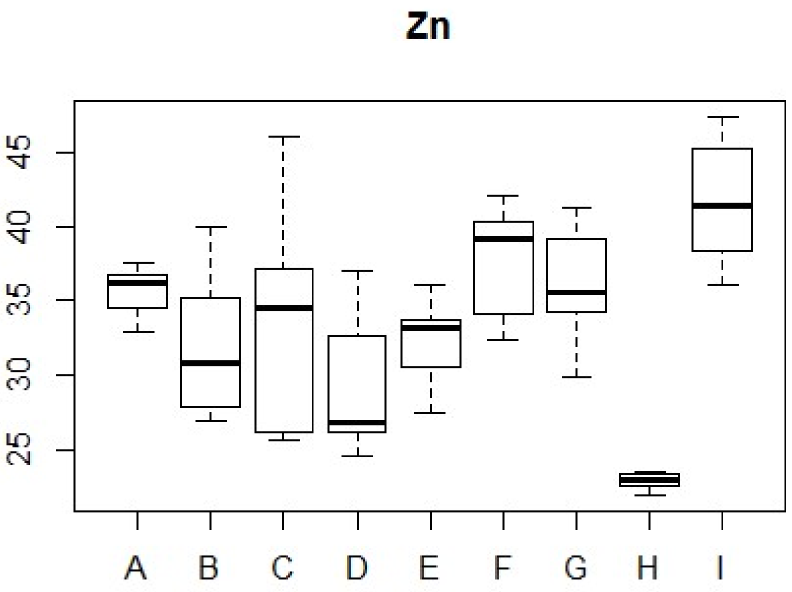
Zn
25
30
35
40
45
A B C D E F G H I

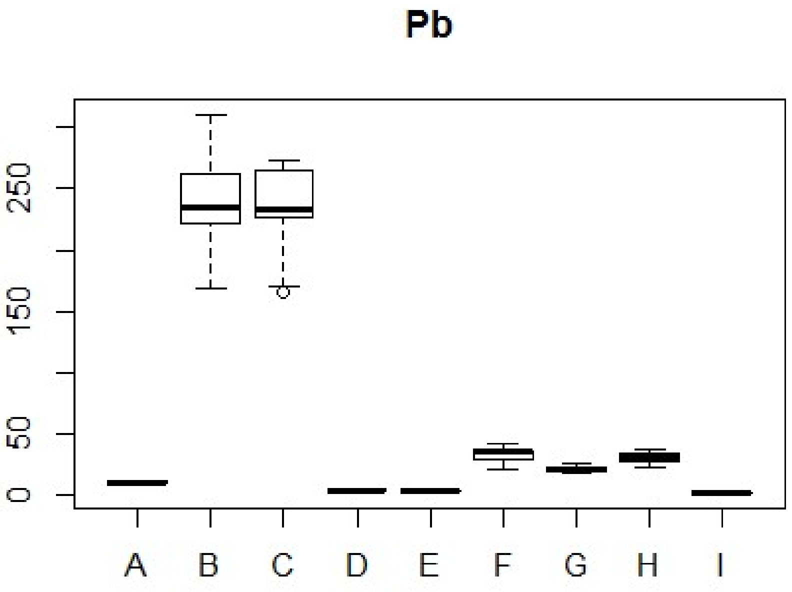
Pb
0
50
150
250
A B C D E F G H I

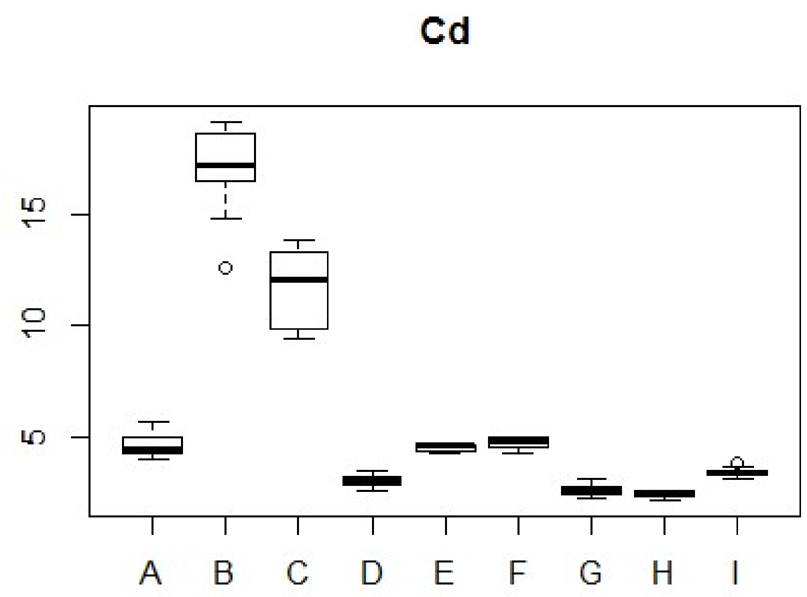

Cd
15
10
5
A B C D E F G H I

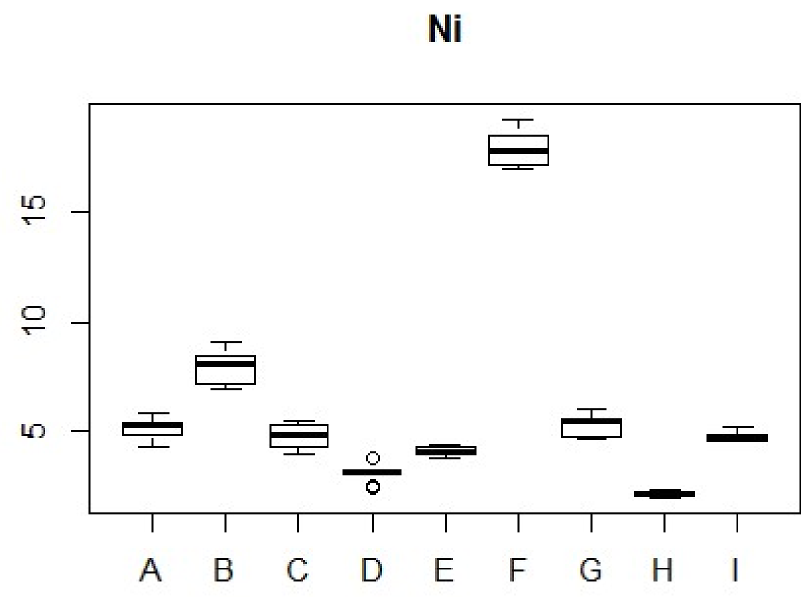

Ni
15
10
5
A B C D E F G H I

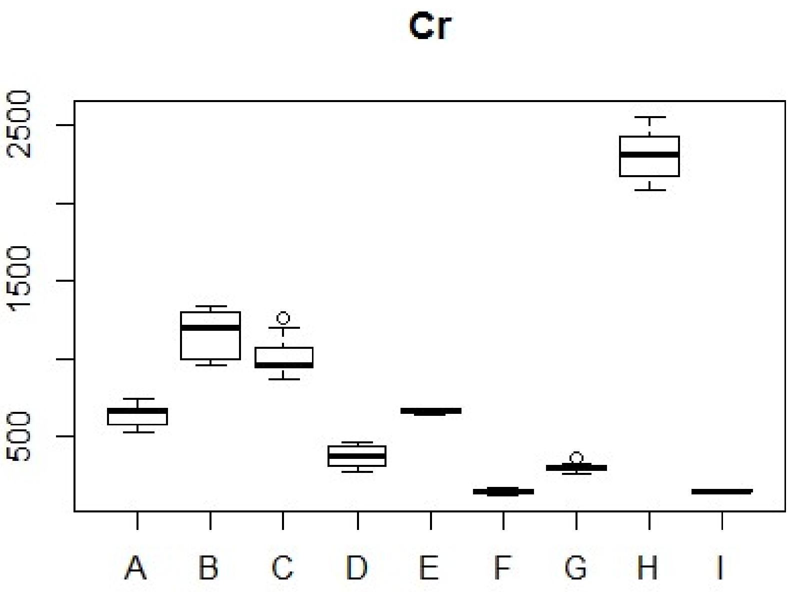
Cr
2500
1500
500
A
B
C
D
E
F
G
H
I

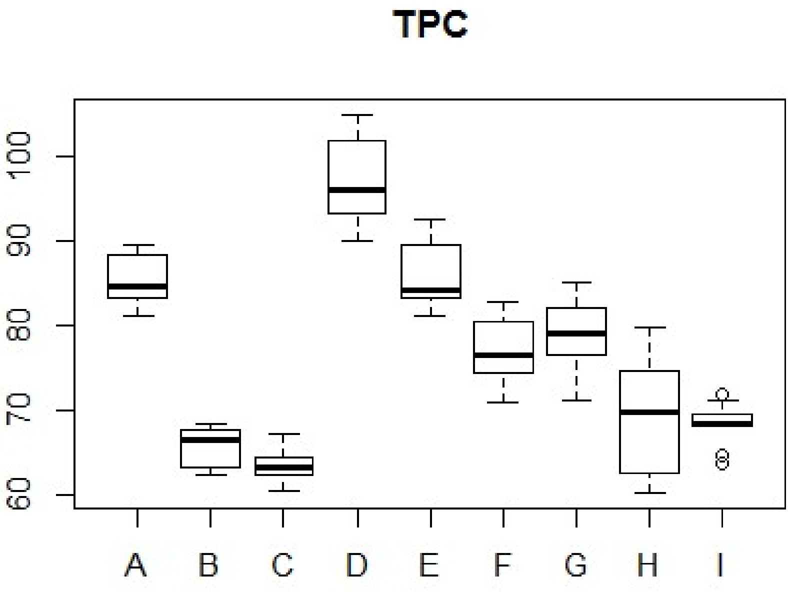
TPC
100
90
80
70
60
A
B
C
D
E
F
G
H
I

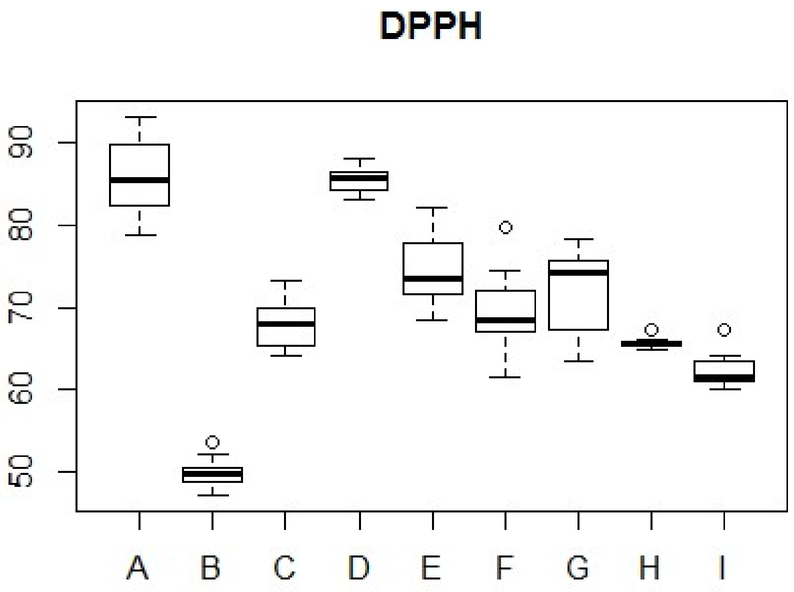
DPPH
90
80
70
60
50
A B C D E F G H I

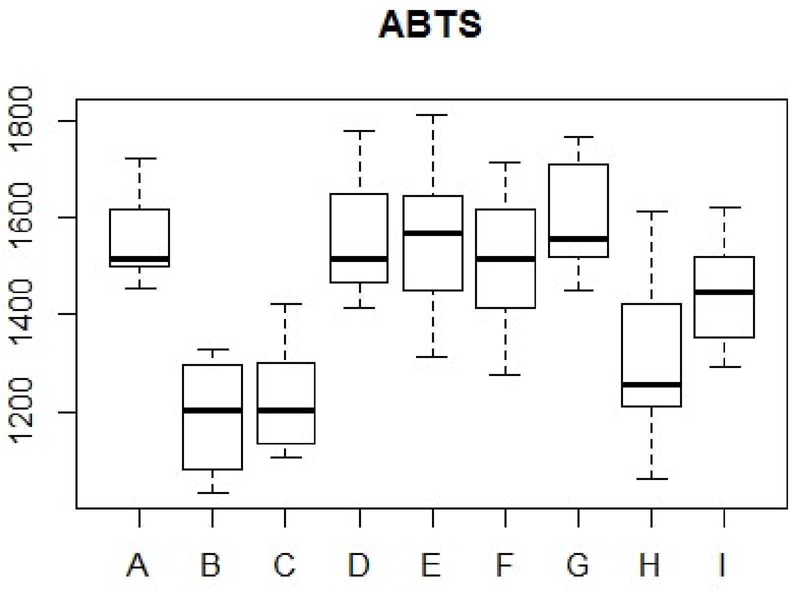
ABTS
1800
1600
1400
1200
A B C D E F G H I
